# AlphaBeta: computational inference of epimutation rates and spectra from high-throughput DNA methylation data in plants

**DOI:** 10.1186/s13059-020-02161-6

**Published:** 2020-10-06

**Authors:** Yadollah Shahryary, Aikaterini Symeonidi, Rashmi R. Hazarika, Johanna Denkena, Talha Mubeen, Brigitte Hofmeister, Thomas van Gurp, Maria Colomé-Tatché, Koen J.F. Verhoeven, Gerald Tuskan, Robert J. Schmitz, Frank Johannes

**Affiliations:** 1 Technical University of Munich, Department of Plant Sciences, Liesel-Beckmann-Str. 2, Freising, 85354 Germany; 2 Technical University of Munich, Institute for Advanced Study, Lichtenbergstr. 2a, Garching, 85748 Germany; 3 Institute of Computational Biology, Helmholtz Zentrum München, Ingolstädter Landstr. 1, Neuherberg, 85764 Germany; 4 Institute of Bioinformatics, 120 East Green Street, Athens, 30602 USA; 5 European Research Institute for the Biology of Ageing, University of Groningen, University Medical Centre Groningen, A. Deusinglaan 1, Groningen, 9713 AV Netherlands; 6 TUM School of Life Sciences Weihenstephan, Technical University of Munich, Emil-Erlenmeyer-Forum 2, Freising, 85354 Germany; 7 Netherlands Institute of Ecology (NIOO-KNAW), Department of Terrestrial Ecology, Wageningen, Wageningen, The Netherlands; 8 The Center for Bioenergy Innovation, Oak Ridge National Laboratory, Oak Ridge, USA; 9 Department of Genetics, The University of Georgia, 120 East Green Street, Athens, 30602 USA

**Keywords:** Epimutation, DNA methylation, Plants, Trees, Epigenetics, Epimutation rate, Evolution, Molecular clock, Epigenetic clock, Bioinformatics software tool, R/Bioconductor package

## Abstract

Stochastic changes in DNA methylation (i.e., spontaneous epimutations) contribute to methylome diversity in plants. Here, we describe *AlphaBeta*, a computational method for estimating the precise rate of such stochastic events using pedigree-based DNA methylation data as input. We demonstrate how *AlphaBeta* can be employed to study transgenerationally heritable epimutations in clonal or sexually derived mutation accumulation lines, as well as somatic epimutations in long-lived perennials. Application of our method to published and new data reveals that spontaneous epimutations accumulate neutrally at the genome-wide scale, originate mainly during somatic development and that they can be used as a molecular clock for age-dating trees.

## Introduction

Cytosine methylation is an important chromatin modification and a pervasive feature of most plant genomes. It has major roles in the silencing of transposable elements (TEs) and repeat sequences and is also involved in the regulation of some genes [1]. Plants methylate cytosines at symmetrical CG and CHG sites, but also extensively at asymmetrical CHH sites, where H= A, T, C. The molecular pathways that establish and maintain methylation in these three sequence contexts are well-characterized [2] and are broadly conserved across plant species [3–7]. Despite its tight regulation, the methylation status of individual cytosines or of clusters of cytosines is not always faithfully maintained across cell divisions. As a result, cytosine methylation is sometimes gained or lost in a stochastic fashion, a phenomenon that has been termed “spontaneous epimutation.” In both animals and plants, spontaneous epimutations have been shown to accumulate throughout development and aging [8], probably as a byproduct of the mitotic replication of small stem cell pools that generate and maintain somatic tissues.

However, in plants, spontaneous epimutations are not only confined to somatic cells, but occasionally pass through the gametes to subsequent generations [9, 10]. In the model plant *Arabidopsis thaliana* (*A. thaliana*), these transgenerationally heritable (i.e., “germline”) epimutations are mainly restricted to CG sites and appear to be absent or not detectable at CHG and CHH sites [11–14]. Initial estimates in *A. thaliana* indicate CG “germline” epimutations are about five orders of magnitude more frequent than genetic mutations (∼10^−4^ vs. ∼10^−9^ per site per haploid genome per generation) [12, 14–16]. Because of these relatively high rates, CG methylation differences accumulate rapidly in the *A. thaliana* genome and generate substantial methylation diversity among individuals in the course of only a few generations [12, 17–19] [20].

**Fig. 1:**
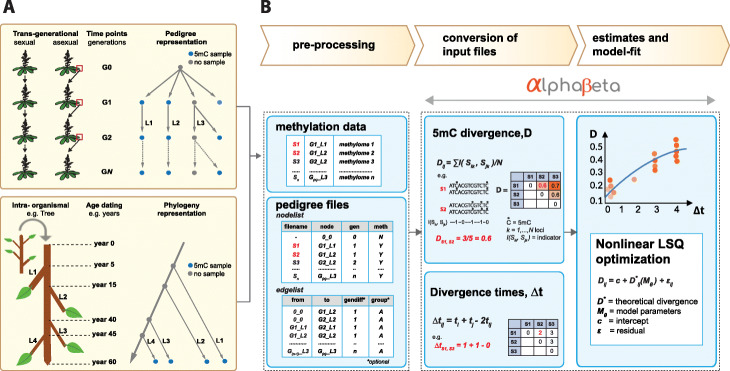
Overview of the AlphaBeta computational pipeline. **a** Top panel: Construction of multi-generational (G0 to GN) mutation accumulation (MA) lines through sexual (selfing or sibling mating) or asexual (clonal) propagation. The different lineages (L1 to L3) can be represented as a pedigree. The pedigree branch point times and the branch lengths are typically known, a priori, from the experimental design. 5mC sampling can be performed at selected generations, either from plant material of direct progenitors or from siblings of those progenitors. The data can be used to estimate the rate and spectrum of “germline” epimutations. Bottom panel: Long-lived perennials, such as trees, can be viewed as a natural mutation accumulation system. The tree branching structure can be treated as an intra-organismal phylogeny of somatic lineages. 5mC samples can be performed on leaf tissues from selected branches. Along with coring data, the leaf methylomes can be used to estimate the rate and spectrum of somatic epimutations. **b** Data pre-processing: *AlphaBeta* requires methylation data and pedigree data as input. File conversion: Using the input files, *AlphaBeta* calculates the 5mC divergence (*D*) as well as divergence time (*Δt*) between all sample pairs. Model estimation: *AlphaBeta* fit competing epimutation models to the data. The model parameters are estimated using numerical non-linear least squares optimization. Model comparisons allow for tests of selection and neutrality

A key experimental challenge in studying epimutational processes in a multi-generational setting is to be able to distinguish “germline” epimutations from other types of methylation changes, such as those associated with segregating genetic variation or transient environmental perturbations [21]. Mutation accumulation (MA) lines grown in controlled laboratory conditions are a powerful experimental system to achieve this. MA lines are derived from a single isogenic founder and are independently propagated for a large number of generations. The lines can be advanced either clonally or sexually, i.e., self-fertilization or sibling mating (Fig. 1a). In clonally produced MA lines, the isogenicity of the founder is not required because the genome is “fixed” due to the lack of genetic segregation.

The kinship among the different MA lineages can be presented as a pedigree (Fig. 1a). The structure (or topology) of these pedigrees is typically known, a priori, as the branch-point times and the branch lengths are deliberately chosen as part of the experimental design. In conjunction with multi-generational methylome measurements, MA lines therefore permit “real-time” observations of “germline” epimutations against a nearly invariant genomic background and can facilitate estimates of the per-generation epimutation rates [11]. Sequenced methylomes from a large number of sexually derived MA lines are currently available in *A. thaliana* [12–14, 18, 22, 23] and rice [24], and various other MA lines are currently under construction for epimutation analysis in different genotypes, environmental conditions, and plant species.

Beyond experimentally derived MA lines, natural mutation accumulation systems can also be found in the context of plant development and aging. An instructive example is long-lived perennials, such as trees, whose branching structure can be interpreted as a pedigree (or phylogeny) of somatic lineages that carry information about the epimutational history of each branch [25]. In this case, the branch-point times and the branch lengths can be determined ad hoc using coring data or other types of dating methods (Fig. 1a). By combining this information with contemporary leaf methylome measurements, it is possible to infer the rate of somatic epimutations as a function of age (see also co-submission, [26]).

Attempts to infer the rate of spontaneous epimutations in these diverse plant systems are severely hampered by the lack of available analytical tools. Naive approaches that try to count the number of epimutations per some unit of time cannot be used in this setting, because DNA methylation measurements are far too noisy. On the technological side, this noise stems from increased sequencing and alignment errors of bisulphite reads and bisulphite conversion inefficiencies. On the biological side, increased measurement error may result from within-tissue heterogeneity in 5mC patterns [27] and the fact that DNA methylomes are in part transcriptionally responsive to variation in environmental/laboratory conditions [28]. To overcome these challenges, we previously implemented a model-based estimation method, which was originally designed for the analysis of selfing-derived mutation accumulation lines [12]. This approach appropriately accounts for measurement error in the data by describing the time-dependent accumulation of epimutations through an explicit statistical model (Fig. 1b). Fitting this model to pedigree-based 5mC measurements yields estimates of the rate of spontaneous methylation gains and losses and provides a quantitative basis for predicting DNA methylation dynamics over time.

Here, we generalize this method and present *AlphaBeta*, the first software package for inferring the rate and spectrum of “germline” and somatic epimutations in plants. *AlphaBeta* can be widely applied to multi-generational data from sexually or asexually derived MA lines, as well as to intra-generational data from long-lived perennials such as trees. Drawing on novel and published data, we demonstrate the power and versatility of our approach and make recommendations regarding its implementation.

## The AlphaBeta method

We start from the assumption that 5mC measurements have been obtained from multiple sampling time-points throughout the pedigree. These measurements can come from whole genome bisulphite sequencing (WGBS) [29] [30], reduced representation bisulphite sequencing (RRBS) [31], or epigenotyping-by-sequencing (epiGBS) [32] technologies, and possibly also from array-based methods. We only require that a “sufficiently large” number of loci has been measured. Moreover, with multigenerational data, we allow measurements to come from plant material of direct progenitors, or else from individual or pooled siblings of those progenitors (Fig. 1a).

### Calculating 5mC divergence

For the *i*th sequenced sample in the pedigree, let *s*_*ik*_ be the observed methylation state at the *k*th locus (*k*=1⋯*N*). Here, the *N* loci can be individual cytosines or pre-defined regions (i.e., cluster of cytosines). We assume that *s*_*ik*_ takes values 1, 0.5, or 0, according to whether the diploid epigenotype at that locus is *m*/*m*,*m*/*u*,*u*/*u*, respectively, where *m* is a methylated and *u* is an unmethylated epiallele. Using this coding, we calculate the mean absolute 5mC divergence, *D*, between any two samples *i* and *j* in the pedigree as follows:

1 $$\begin{array}{@{}rcl@{}} D_{ij} = \sum_{k=1}^{N} I(s_{ik}, s_{jk})N^{-1}, \end{array} $$

where *I*(·) is an indicator function, such that

$$I(s_{ik}, s_{jk})= \left\{ \begin{array}{ll} 0 \text{ if} s_{ik} = s_{jk} \\ \frac{1}{2} \text{ if} s_{ik} = 0.5 \text{ and} s_{jk} \in \{0,1\} \\ \frac{1}{2} \text{ if} s_{jk} = 0.5 \text{ and} s_{ik} \in \{0,1\} \\ 1 \text{ if} s_{ik} = 0 \text{ and} s_{jk} = 1 \\ 1 \text{ if} s_{jk} = 1 \text{ and} s_{ik} = 0. \\ \end{array} \right. $$

The software automatically calculates *D*_*ij*_ and *Δt* for all unique sample pairs using as input the methylation state calls and the pedigree coordinates of each sample (Fig. 1b).

### Modelling 5mC divergence

We model the 5mC divergence as

2 $$\begin{array}{@{}rcl@{}} D_{ij} = c + D^{\bullet}_{ij}(M_{\Theta}) + \epsilon_{ij}. \end{array} $$

Here, *ε*_*ij*_∼*N*(0,*σ*^2^) is the normally distributed residual error, *c* is the intercept, and $D^{\bullet }_{ij}(M_{\Theta })$ is the expected divergence between samples *i* and *j* as a function of an underlying epimutation model *M*(·) with parameter vector *Θ* (see below). We have that

$$\begin{array}{@{}rcl@{}} D^{\bullet}_{ij}(M_{\Theta}) &=& \sum_{n\in v} \sum_{l\in v} \sum_{m\in v} I(l,m) \\ &\cdot& Pr(s_{ik}=l, s_{jk}=m|s_{ijk}=n,M_{\Theta})\\ &\cdot& Pr(s_{ijk}=n|M_{\Theta}), \end{array} $$

where *s*_*ijk*_ is the methylation state at the *kth* locus of the most recent common ancestor of samples *i* and *j*, and *v*={0,0.5,1}. Since samples *s*_*i*_ and *s*_*j*_ are conditionally independent, we can further write:

$$\begin{array}{@{}rcl@{}} Pr(s_{ik}, s_{jk}|s_{ijk},M_{\Theta}) &=& Pr(s_{ik}|s_{ijk},M_{\Theta}) \\ &\cdot& Pr(s_{jk}|s_{ijk},M_{\Theta}). \end{array} $$

To be able to evaluate these conditional probabilities, it is necessary to posit an explicit form for the epimutational model, *M*_*Θ*_. To motivate this, we define **G** to be a 3×3 transition matrix, which summarizes the probability of transitioning from epigenotype *l* to *m* in the time interval [*t*,*t*+1]:

$$\begin{array}{*{20}l} &\qquad u/u (t+1) {\kern5pt} m/u (t+1) {\kern10pt} m/m (t+1)\\ \mathbf{G}&= \left[\begin{array}{ccc} f_{11}(\alpha, \beta, w) & f_{12}(\alpha, \beta, w) & \cdot\\ f_{21}(\alpha, \beta, w) & \cdot & \cdot \\ \cdot & \cdot & f_{33}(\alpha, \beta, w) \\ \end{array}\right] \begin{array}{c} u/u \;(t)\\ m/u \,(t)\\ m/m (t) \end{array} \end{array} $$

The elements of this matrix are a function of gain rate *α* (i.e., the probability of a stochastic epiallelic switch from an unmethylated to a methylated state within interval [*t*,*t*+1]), the loss rate *β* (i.e., the probability of a stochastic epiallelic switch from a methylated to an unmethylated state), and the selection coefficient *w* (*w*∈[0,1]). It can be shown that for a diploid system propagated by selfing, **G** has the form

$$\left[\begin{array}{ccc} (1-\alpha)^{2} & 2(1-\alpha)\alpha & \alpha^{2} \\ \frac{1}{4}(\beta+1-\alpha)^{2} & \frac{1}{2}(\beta+1-\alpha)(\alpha+1-\beta) & \frac{1}{4}(\alpha+1-\beta)^{2} \\ \beta^{2} & 2(1-\beta)\beta & (1-\beta)^{2} \end{array}\right]\circ \; \mathbf{W}, $$

and for systems that are propagated clonally or somatically **G** is:

$$\left[\begin{array}{ccc} (1-\alpha)^{2} & 2(1-\alpha)\alpha & \alpha^{2} \\ \beta(1-\alpha) & (1-\alpha)(1-\beta)+\alpha\beta & \alpha(1-\beta)\\ \beta^{2} & 2(1-\beta)\beta & (1-\beta)^{2} \end{array}\right] \circ \; \mathbf{W}, $$

where ∘ is the Hadamard product and **W** is a matrix of selection coefficients of the form

$$\left[\begin{array}{ccc} w & \frac{(w+1)}{2} & 1 \\ w & \frac{(w+1)}{2} & 1 \\ w & \frac{(w+1)}{2} & 1 \\ \end{array}\right] \text{ or } \left[\begin{array}{ccc} 1 & \frac{(w+1)}{2} & w \\ 1 & \frac{(w+1)}{2} & w \\ 1 & \frac{(w+1)}{2} & w \\ \end{array}\right] $$

depending on whether selection is against epiallele *u* or *m*, respectively.

Using this formalism, we can distinguish four different models, which we denote by *ABneutral*, *ABmm*, *ABuu*, and *ABnull*. Model *ABneutral* assumes that the accumulation of spontaneous 5mC gains and losses is selectively neutral (*w*=1,*α* and/or *β*>0). In this special case, all epigenotype transitions from time *t* to *t*+1 are only governed by the rates *α* and *β*, and—in the case of selfing—also by the Mendelian segregation of epialleles *u* and *m*. The selection models *ABmm* and *ABuu*, by contrast, assume that epimutation accumulation is in part shaped by selection against spontaneous losses or gains of 5mC, respectively (0≤*w*<1,*α* and/or *β*>0). For example, with selection in favor of epiallele *u* (model *ABuu*), the fitness of epihomozygote *m*/*m* and epiheterozygote *m*/*u* are reduced by a factor of *w* and (*w*+1)/2, respectively. We incorporate this fitness loss directly into the transition matrix by weighing the transition probabilities to these epigenotypes accordingly [33]. Similar arguments hold for the case where selection is for epiallele *m*. As a reference, we define model *ABnull* as the null model of no accumulation, with *α*=0,*β*=0, and *w*=1.

To ensure that the rows of **G** (i.e., the transition probabilities) still sum to unity in the presence of selection, we redefine **G** using the normalization:

$$\mathbf{G^{\prime}} = \left[\begin{array}{ccc} (\sum_{i} \mathbf{G}_{1i})^{-1} & 0 & 0 \\ 0 & (\sum_{i} \mathbf{G}_{2i})^{-1} & 0 \\ 0 & 0 & (\sum_{i} \mathbf{G}_{3i})^{-1} \\ \end{array}\right] \cdot \mathbf{G} $$

Based on Markov chain theory, the conditional probability *Pr*(*s*_*ik*_|*s*_*ijk*_,*M*_*Θ*_) can then be expressed in terms of *G*^′^ as follows:

$$\begin{array}{@{}rcl@{}} {\sum_{n}} Pr(s_{ik}=0|s_{ijk}=n, M_{\Theta}) = \sum_{r=1}^{3}(\mathbf{G^{\prime}}^{t_{i} - t_{ij}})_{r1} \\ {\sum_{n}} Pr(s_{ik}=0.5|s_{ijk}=n, M_{\Theta}) = \sum_{r=1}^{3}(\mathbf{G^{\prime}}^{t_{i} - t_{ij}})_{r2} \\ {\sum_{n}} Pr(s_{ik}=1|s_{ijk}=n, M_{\Theta}) = \sum_{r=1}^{3}(\mathbf{G^{\prime}}^{t_{i} - t_{ij}})_{r3} \end{array} $$

where *t*_*i*_ is the time-point corresponding to sample *i* and *t*_*ij*_ is the time-point of the most recent common ancestor shared between samples *i* and *j*, (*t*_*ij*_≤*t*_*i*_,*t*_*j*_), and *r* is a row index. Expressions for *Pr*(*s*_*jk*_|*s*_*ijk*_,*M*_*Θ*_,*t*_*j*_) can be derived accordingly, by simply replacing *t*_*i*_ by *t*_*j*_ in the above equation. Note that the calculation of these conditional probabilities requires repeated matrix multiplication. However, a direct evaluation of these equations is also possible using the fact that

$$\begin{array}{@{}rcl@{}} \mathbf{G^{\prime}}^{t_{i} - t_{ij}} = \mathbf{p}\mathbf{V}^{t_{i} - t_{ij}}\mathbf{p}^{-1} \text{ and } \mathbf{G^{\prime}}^{t_{j} - t_{ij}} = \mathbf{p}\mathbf{V}^{t_{j} - t_{ij}}\mathbf{p}^{-1}, \end{array} $$

where **p** is the eigenvector of matrix *G*^′^ and **V** is a diagonal matrix of eigenvalues. For selfing and clonal/somatic systems, these eigenvalues and eigenvectors can be obtained analytically.

Finally, to derive $D^{\bullet }_{ij}(M_{\Theta })$, we also need to supply *Pr*(*s*_*ijk*_=*n*|*M*_*Θ*_); that is, the probability that locus *k* in the most recent common ancestor of samples *i* and *j* is in state *n* (*n*∈{0,0.5,1}). To do this, consider the methylome of the pedigree founder at time *t*=1, and let **π**=[*p*_1_ *p*_2_ *p*_3_] be a row vector of probabilities corresponding to states *u*/*u*,*u*/*m* and *m*/*m*, respectively. Using Markov Chain theory, we have

$$\begin{array}{@{}rcl@{}} Pr(s_{ijk}= 0|M_{\Theta}) &=& \left[ \pi \, \mathbf{G^{\prime}}^{(t_{ij}-1)} \right]_{1} \\ Pr(s_{ijk}= 0.5|M_{\Theta}) &=& \left[ \pi \, \mathbf{G^{\prime}}^{(t_{ij}-1)} \right]_{2} \\ Pr(s_{ijk}= 1|M_{\Theta}) &=& \left[ \pi \, \mathbf{G^{\prime}}^{(t_{ij}-1)} \right]_{3} \end{array} $$

In many situations, the most recent common ancestor happens to be the pedigree founder itself, so that *t*_*ij*_=1. In the case where the methylome of the pedigree founder has been measured, the probabilities *p*_1_,*p*_2_ and *p*_3_ can be estimated directly from the data using *x*_1_*N*^−1^,*x*_2_*N*^−1^ and *x*_3_*N*^−1^, respectively. Here, *x*_1_,*x*_2_, and *x*_3_ are number of loci that are observed to be in states *u*/*u*,*u*/*m*,*m*/*m*, and *N* is the total number of loci. Typically, however, *x*_2_ is unknown as most DMP and DMR callers do not output epiheterozygous states (i.e., intermediate methylation calls). Instead, we therefore use

$$\begin{array}{@{}rcl@{}} p_{1} = \frac{x_{1}}{N},\qquad p_{2} =\gamma \frac{x_{3}}{N},\qquad p_{3} = (1-\gamma) \frac{x_{3}}{N} \end{array} $$

where *γ*∈[0,1] is an unknown parameter.

### Model inference

To obtain estimates for *Θ*, we seek to minimize the least-squares using

3 $$\begin{array}{@{}rcl@{}} \nabla {\sum_{q=1}^{M}} \left(D_{q} - D^{\bullet}_{q}(M_{\Theta}) - c \right)^{2} = \mathbf{0}, \end{array} $$

where the summation is over all *M* unique pairs of sequenced samples in the pedigree. Minimization is performed using the “Nelder-Mead” algorithm as part of the optimx package in R. However, from our experience, convergence is not always stable, probably because the function $D^{\bullet }_{q}(M_{\Theta })$ is complex and highly non-linear. We therefore include the following minimization constraint:

4 $$\begin{array}{*{20}l} \nabla {\sum_{q=1}^{M}} \left(D_{q} - D^{\bullet}_{q}(M_{\Theta}) - c \right)^{2} \end{array} $$

5 $$\begin{array}{*{20}l} + M\left(\tilde{p_{1}} - p_{1}(t_{\infty}, M_{\Theta}) \right)^{2} = \mathbf{0}. \end{array} $$

Here, *p*_1_(*t*_*∞*_,*M*_*Θ*_) is the equilibrium proportion of *u*/*u* loci in the genome as *t*→*∞*. For a selfing system with *w*=1, we have that

$$\begin{array}{@{}rcl@{}} p_{1}(t_{\infty}, M_{\Theta}) =\frac{\beta((1-\beta)^{2} - (1-\alpha)^{2} -1)}{(\alpha + \beta)((\alpha + \beta -1)^{2} - 2)}, \end{array} $$

and for a clonal/somatic system, it is:

$$\begin{array}{@{}rcl@{}} p_{1}(t_{\infty}, M_{\Theta}) = \frac{\beta^{2}}{(\alpha+\beta)^{2}}. \end{array} $$

For the case where 0≤*w*<1, the equations are more complex and are omitted here. Note that the value $\tilde {p_{1}}$ is an empirical guess at these equilibrium proportions. For samples whose methylomes can be assumed to be at equilibrium, we have that *p*_1_(*t*=1)=*p*_1_(*t*=2)=⋯=*p*1(*t*_*∞*_), meaning that the proportion of loci in the genome that are in state *u*/*u* are (dynamically) stable for any time *t*. Under this assumption, $\tilde {p_{1}}$ can be replaced by $\overline {p_{1}}$, which is the average proportion of *u*/*u* loci calculated from all pedigree samples.

### Confidence intervals

We obtain confidence intervals for the estimated model parameters by boostrapping the model residuals. The procedure has the following steps: (1) For the *q*th sample pair *q* (*q*=1,⋯,*M*), we define a new response variable $B_{q} = \hat {D_{q}} + \hat {\epsilon }_{k}$, where $\hat {D_{q}}$ is the fitted divergence for the *q*th pair and $\hat {\epsilon }_{k}$ is drawn at random and with replacement from the 1×*M* vector of fitted model residuals. (2) Refit the model using the new response variable and obtain estimates for the model parameters. (3) Repeat steps 1 to 2 a large number of times to obtain a bootstrap distribution. (4) Use the bootstrap distribution from 3 to obtain empirical confidence intervals.

### Testing for selection

To assess whether a selection model provides a significantly better fit to the data compared to a neutral model, we define

$$\begin{array}{@{}rcl@{}} RSS_{F}=\sum^{M}_{q=1} \epsilon_{q}(\hat{\Theta})^{2} \end{array} $$

and

$$\begin{array}{@{}rcl@{}} RSS_{R}= \sum^{M}_{q=1} \epsilon_{q}(\hat{\alpha}, \hat{\beta}, \hat{\gamma}, \hat{c} | w=1)^{2} \end{array} $$

to be the estimated residual sums of squares of the full model and reduced (i.e., neutral) model, respectively, with corresponding degrees of freedom *df*_*F*_ and *df*_*R*_. To test for selection, we evaluate the following *F*-statistic:

$$\begin{array}{@{}rcl@{}} F = \frac{(RSS_{R} - RSS_{F})}{RSS_{F}} \cdot \frac{df_{F}}{df_{N}}, \end{array} $$

where *df*_*N*_=*df*_*F*_−*df*_*R*_. Under the Null *F*∼*F*(*df*_*N*_,*df*_*F*_).

## Application

To illustrate the utility of our method, we used *AlphaBeta* to study “germline” epimutations in selfing- and asexually derived MA lines of *Arabidopsis*(*A. thaliana*) and dandelion (*Taraxacum officinale*), as well as somatic epimutations in a single poplar tree (*Populus trichocarpa*). Our goal was to demonstrate the wide range of application of our method and to highlight several novel insights into the nature of spontaneous epimutations in plants.

### Analysis of spontaneous epimutations in selfing-derived *A. thaliana* MA lines

**Fig. 2:**
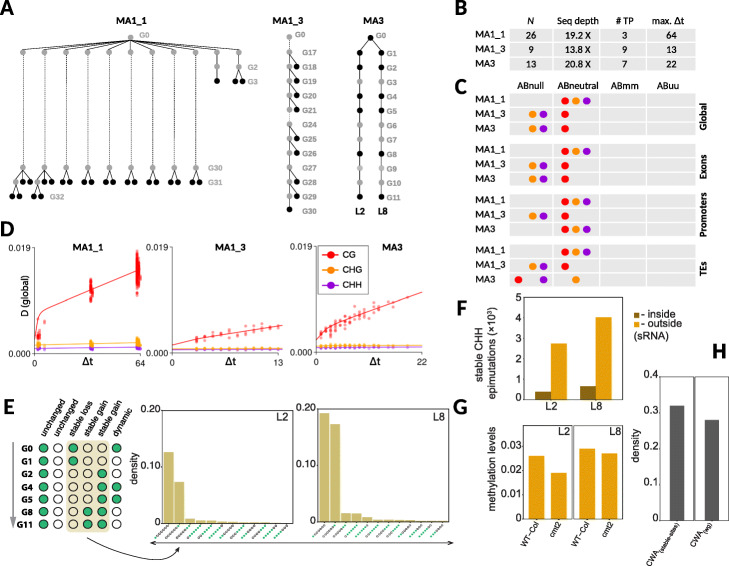
Analysis of “germline” epimutations in *A. thaliana*mutation accumulation (MA) lines. **a** Three different MA pedigrees were analyzed. All three pedigrees were derived from a single Columbia (Col-0) inbred genotype. Two of the pedigrees were previously published (MA1_1, Becker et al. 2011; MA1_3, van der Graaf et al. 2015), and one pedigree (MA3) is new. These three MA pedigrees were chosen because they differ in their topologies, 5mC measurement strategies, and the temporal resolution of the 5mC samples. **b** Overview of the data: *N* is the total number of sequenced samples; Seq depth is the average sequence depths of the samples; # TP is the number of unique time-points (or generations) that are sampled; max. (*Δ*t) is the maximum divergence time (in generations) in the pedigree. **c** Application of models Abnull, Abneutral, ABmm, and Abuu. The best fitting model is indicated for each MA pedigree, sequence context (CG, CHG, and CHH), and genomic feature (global, exons, promoters, TEs). **d** Shown are the fits of the best fitting models for each pedigree and context. **e** Schematic representation of transgenerationally stable CHH epimutations. The barplots indicate the density of stable CHH epimutations in lineages L2 and L8 of the MA3 pedigree. **f** CHH sites featuring stable epimutations tend to fall outside of sRNA clusters in lineages L2 and L8. **g** Analysis of *cmt2* mutant and Col-0 wt from Stroud et al. [2] show loss of methylation in the mutant at the stable CHH epimutation sites, indicating that these loci are targeted by CMT2. **e** Compared to the whole genome (wg), stable CHH loci with stable epimutations are enriched for CWA trinucleotides, which is a preferred substrate for CMT2 binding

We first analyzed three *A. thaliana* MA pedigrees (MA1_1, MA1_3, MA3, see Fig. 2a). We chose these MA pedigrees because they differ markedly in their topologies, 5mC sampling strategies, sequencing method, and depth (Fig. 2a, b, Additional file 1: Table S1). All MA pedigrees were derived from a single Col-0 founder accession. The first MA pedigree (MA1_1) was originally published by Becker et al. [13]. The pedigree data consists of 11 independent lineages with sparsely collected WGBS samples (∼ 19.2X coverage) from generations 3, 31, and 32, and a maximum divergence time (*Δt*) of 64 generations. MA1_3 was previously published by van der Graaf et al. [12]. This data consists of single lineage with dense MethylC-seq measurements (∼ 13.8X coverage) from generations 18 to 30, and a maximum *Δt* of 13 generations. Finally, we present a new pedigree (MA3), which consists of 2 lineages with dense MethylC-seq measurements (∼ 20.8X coverage) from generations 0 to 11, and a maximum *Δt* of 22 generations. Unlike MA1_1 and MA1_3, MA3 has 5mC measurements from progenitor plants of each sampled generation, rather than from siblings of those progenitors (Fig. 2a). Further information regarding the samples, sequencing depths, and platforms is provided in Additional file 1: Table S1. A detailed description of data pre-processing and methylation state calling can be found in the “Materials and data pre-processing” section.

#### Spontaneous epimutations accumulate neutrally over generations

We started by plotting genome-wide (global) 5mC divergence (*D*) against divergence time (*Δt*). *D* increases as a function of *Δt* in all pedigrees (Fig. 2d). A characteristic pattern is the rapid, non-linear increase in *D* for the first ∼ 8 generations followed by a nearly linear increase. As pointed out before [12], the initial non-linearity is driven by the stable segregation and fixation of epiheterozygote loci that originate from the pedigree founder, a phenomenon that has been well-described in the classical genetic theory of experimental line crosses [34–37]. By contrast, the subsequent linear increase in *D* is mainly due to the accumulation of new epimutations that arise de novo during inbreeding. The co-occurrence of these two processes is restricted to mutation accumulation systems that are propagated sexually. In clonally or asexually derived MA lines, the non-linear increase in *D* should be absent, as can indeed be seen in our later analysis of poplar and dandelion (see below).

Another striking insight from the 5mC divergence patterns is that the increase in *D* is particularly pronounced for context CG but appears to be low, or even absent, at CHG and CHH loci. Similar observations have previously led to the hypothesis that the inheritance of spontaneous epimutations may be restricted to CG dinucleotides [11, 12], perhaps as a consequence of the preferential reinforcement of CHG and CHH methylation during sexual reproduction [38, 39]. Using heuristic arguments, it had been further suggested that CG epimutations accumulate neutrally, at least at the level of individual cytosines, meaning that 5mC gains and loss in this context are under no selective constraints [12]. However, these hypotheses have never been tested explicitly due to a lack of analytical tools.

To address this, we fitted models *ABneutral*, *ABmm*, *ABuu*, and *ABnull* to the divergence data of each pedigree (Fig. 2c). As mentioned above (see the “The AlphaBeta method” section), model *ABneutral* assumes that spontaneous 5mC gains and losses accumulate neutrally across generations, *ABmm* assumes that the accumulation is partly shaped by selection against spontaneous losses of 5mC, *ABuu* assumes that the accumulation is partly shaped selection against spontaneous gains, and *ABnull* is the null model of no accumulation. Formal model comparisons revealed that *ABneutral* provides the best fit to the 5mC divergence data in context CG in all pedigrees (Fig. 2c, Additional files 2, 3, and 4: Tables S2-S4). This was true at the genome-wide scale (global) as well as at the sub-genomic scale (exons, promoters, TEs). Globally, *ABneutral* explained between 77 and 90% of the total variance in *D*, indicating that a neutral epimutation model provides a good and sufficient description of the molecular process that generates heritable 5mC changes at level of individual cytosines over time. Interestingly, we also detected, for the first time, highly significant accumulation of neutral epimutations in contexts CHG and CHH (Fig. 2c, Additional files 2, 3, and 4: Tables S2-S4). However, the detection of these accumulation patterns was mainly restricted to MA1_1, the largest of the three pedigrees in terms of both sample size (*N*=26) and divergence times (max. *Δt*=64), and to some extent also to MA3, the second largest of the three pedigrees (*N*=13, max. *Δt*=22).

The detected accumulation of CHH epimutations was somewhat surprising, given that cytosine methylation in this context is typically targeted by the RNA-directed DNA methylation pathway (RdDM). The de novo action of this pathway should prevent the formation of trans-generationally stable epimutations, particularly those originating from DNA methylation loss [40]. To explore this observation in more detail, we inspected specific CHH sites that showed stable methylation status changes over generation time (Fig. 2e). Our analysis revealed that these CHH sites actually fall outside of known sRNA clusters and are therefore unlikely involved in RdDM (Fig. 2f). Instead, they appear to be targeted by CHROMOMETHYLASE 2 (CMT2), an enzyme that maintains methylation at a subset of CHG and CHH sites, independently of RdDM. Support for this hypothesis comes from the fact that these CHH sites are enriched for trinucleotide context CWA (W = A, T) (Fig. 2g), which is a preferred substrate for CMT2 binding [41]. Moreover, a re-analysis of a *cmt2* methylation mutant from Stroud et al. [2] revealed a marked reduction in cytosine methylation at these CHH sites relative to wt (Fig. 2h), providing additional evidence for a maintenance role of CMT2 at these loci.

Taken together, these results provide a possible molecular explanation for the accumulation of CHH epimutations over generation time, at least for specific CHH subcontexts. However, the ability to consistently detect these accumulation patterns from multi-generational pedigree data should be explored more systematically in future studies, particularly as a function of sample size, divergence time, and measurement uncertainly in 5mC divergence.

#### The rate and spectrum of spontaneous CG, CHG, and CHH epimutations

**Fig. 3:**
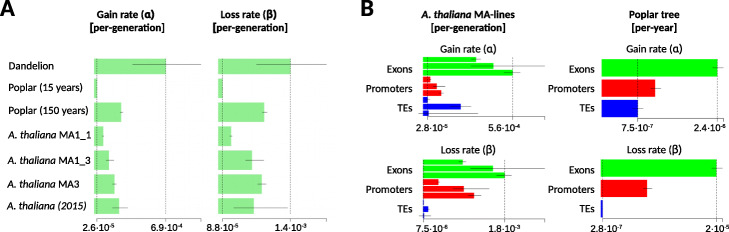
Comparisons of the CG epimutation rates and spectra. **a** Shown are the estimates (± 95% confidence intervals) of the genome-wide (global) CG epimutation rates for the different pedigrees. For comparison, we also show the range of previous estimates from *A. thaliana* MA lines (*A. thaliana* (2015)); see van der Graaf et al. (2015). In poplar, the estimated per-year epimutation rates were converted to per-generation rates by assuming a generation time of 15 years and 150 years. The gain and loss rates are all well within one order of magnitude of each other, and differences are mostly within estimation error. The dashed vertical lines mark off the lower and upper range of the point estimates. **b** Side-by-side comparison of “germlines” and somatic epimutation rate estimates (± 95% confidence intervals) in *A. thaliana* MA lines and poplar, respectively, for selected genomic features. The rank ordering of the magnitude of these rates is similar. For *A. thaliana*, the order of presentation of the pedigrees is MA3, MA1_3, and MA1_1 (from bottom to top within each feature). Feature-specific rates could not be obtained in dandelion since no annotated assembly is currently available

We examined the estimated epimutation rates corresponding to the best fitting models from above (Fig. 3a, Additional files 2, 3, and 4: Tables S2-S4). Globally, we found that the CG methylation gain rate (*α*) is 1.4·10^−4^ per CG per haploid genome per generation on average (range 8.6·10^−5^ to 1.94·10^−4^) and the loss rate (*β*) is 5.7·10^−4^ on average (range 2.5·10^−4^ to 8.3·10^−4^). Using data from pedigree MA1_1, we also obtained the first epimutation rate estimates for contexts CHG and CHH. The gain and loss rates for CHG were 3.5·10^−6^ and 5.8·10^−5^ per CHG per haploid genome per generation, respectively; and for CHH, they were 1.9·10^−6^ and 1.6·10^−4^ per CHH per haploid genome per generation. Hence, transgenerationally heritable CHG and CHH epimutations arise at rates that are about 1 to 2 orders of magnitude lower than CG epimutations in *A. thaliana*, which is reflected in the relatively slow increase of 5mC divergence in non-CG contexts over generation time (Fig. 2d).

In addition to global estimates, we also assessed the gain and loss rates for selected genomic features (exons, promoters, TEs). In line with previous analyses [12], we found striking and consistent rate differences, with exon-specific epimutation rates being 2 to 3 orders of magnitude higher than TE-specific rates (Fig. 3b, Additional files 2, 3, and 4: Tables S2-S4). Interestingly, this trend was not only restricted to CG sites, but was also present in contexts CHG and CHH. This later finding points to yet unknown sequence or chromatin determinants that affect the 5mC fidelity of specific regions across cell divisions, independently of CG, CHG, and CHH methylation pathways.

We note that the CG epimutation rates reported here differ slightly from our previous estimates [12] (Fig. 3a, Additional files 3 and 4: Tables S3-S4). This small discrepancy is mainly the result of differences in the data pre-processing. Application of *AlphaBeta* to published pre-processed samples yielded similar results to those reported previously (data not shown), indicating that the statistical inference itself is consistent. Unlike past approaches, we here utilized the recent *MethylStar* pipeline [42] for data pre-processing and methylation state calling. The use of this pipeline leads to a substantial increase in the number of high-confidence cytosine methylation calls for downstream epimutation analysis (Additional file 5: Table S5). This boost in sample size is reflected in the lower variation in *α* and *β* estimates across MA pedigree compared with previous reports [12] (Fig. 3a, Additional files 2 and 3: Tables S2-S3).

### Analysis of spontaneous somatic epimutations in poplar

Despite the above quantitative insights into the rate and spectrum of spontaneous epimutation in *A. thaliana*, it remains unclear how and where these epimutations actually originate in the plant life cycle. One hypothesis is that they are the result of imperfect 5mC maintenance during the mitotic replication of meristematic cells which give rise to all above and below ground tissues, including the “germline” (Additional file 6: Figure S1). As the germline is believed to be derived quite late in development from somatic precursors, somatic epimutations that accumulate during aging can subsequently be passed to offspring. An alternative hypothesis is that heritable epimutations originate as a byproduct of sRNA-mediated reinforcement errors in the sexual cell linages. One way to distinguish these two possibilities is to study epimutational processes in systems that bypass or exclude sexual reproduction.

Long-lived perennials, such as trees, represent a powerful system to explore this. A tree’s branching structure can be interpreted as an intra-organismal phylogeny of different somatic cell lineages. It is therefore possible to track mutations and epimutations and their patterns of inheritances across different tree sectors. Recently, there has been a surge of interest in characterizing somatic nucleotide mutations in trees using whole genome sequencing data [43–46]. These studies have shown that fixed mutations arise sequentially in different tree sectors, thus pointing at a shared meristematic origin.

**Fig. 4:**
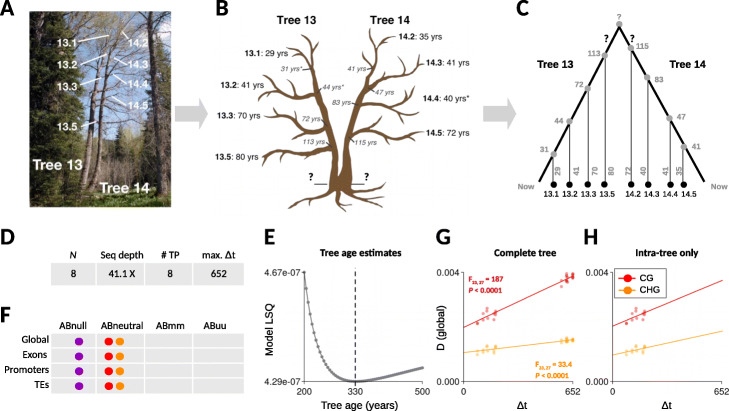
Analysis of somatic epimutations in poplar. **a** A single poplar (*P. trichocarpa*) tree was analyzed. Tree 13 and 14 are two main stems that have diverged early in development. Four branches from each tree were chosen and aged by coring. **b** Shown are the coring sites along with the coring-based branch ages. Age coring proved technically challenging at the bottom of the tree and led to unintelligible ring counts. An educated guess places the age of the tree between 250 and 350 years. **c** The tree can be presented as an intra-organismal phylogeny. Leaf methylomes were collected from each of the selected branches and served as input for *AlphaBeta*. **d** Overview of the data: *N* is the total number of sequenced samples; Seq depth is the average sequence depths of the samples; # TP is the number of unique time-points that are sampled; max. (*Δt*) is the maximum divergence time (in years) between leaf samples. **e***AlphaBeta* was fitted to the global CG methylation divergence data of the complete tree data treating tree age as an unknown parameter. Model residual (LSQ) was minimized at an age of 330 years, which is our estimate of the age of the tree. **f** Model comparisons indicate that somatic epimutations accumulate neutrally in context CG (red) and CHG (orange) during aging, both at the global scale as well as within specific genomic features (exons, promoters, TEs). **g**, **h** Shown are the fits of model *ABneutral* to the global CG (red) and CHG (orange) methylation divergence data of the complete tree (intra-tree + inter-tree, **g**), as well as for tree 13 and tree 14 separately (intra-tree, **h**)

To facilitate the first insights into epimutational processes in long-lived perennials, we applied *AlphaBeta* to MethylC-seq leaf samples (∼41.1X coverage) from 8 separate branches of a single poplar (*Populus trichocarpa*) tree (see also co-submission, [26]). The tree features two main stems (here referred to as tree 13 and tree 14), which were originally thought to be two separate trees (Fig. 4a, b). However, both stems are stump sprouts off an older tree that was knocked down about 350 years ago. In other words, tree 13 and tree 14 are clones that have independently diverged for a long time. Four branches from each tree were chosen and aged by coring at the points where each branch meets the main stem as well as at the terminal branch (Fig. 4a, b, see the “Materials and data pre-processing” section). Age dating of the bottom sector of the tree proved particularly challenging because of heart rot, rendering estimates of the total tree age imprecise. However, an estimate based on diameter measurements places the minimum age of the tree at about 250 years.

#### Inferring total tree age from leaf methylome data

We used the coring-based age measurements from each of the branches along with the branch points to calculate divergence times (*Δt*) between all pairs of leaf samples (Fig. 4c). We did this by tracing back their ages (in years) along the branches to their most recent common branch point (i.e., “founder cells”) (Additional file 6: Figure S1). The calculation of the divergence times for pairs of leaf samples originating from tree 13 and tree 14 was not possible since the total age of the tree was unknown. To solve this problem, we included the total age of the tree as an additional unknown parameter into our epimutation models. Our model estimates revealed that the total age of the tree is approximately 330 years (Fig. 4e), an estimate that fits remarkably well with the hypothesized age window (between 250 and 350 years). Furthermore, the model fits provided overwhelming evidence that somatic epimutations, in poplar, accumulate in a selectively neutral fashion during aging, both at the genome-wide scale (globally) as well as at the sub-genomic scale (exons, promoters, TEs) (Fig. 4f, see also co-submission [26]). This was true for CG and CHG contexts (Fig. 4g). The fact that the accumulation of CHG epimutations is so clearly detectable in poplar, but only inconsistently in *A. thaliana* MA lines, could indicate that somatically acquired CHG methylation changes experience some level of reprogramming during sexual reproduction. But this hypothesis should be tested more directly using cell-type-specific sequencing approaches. To rule out that the somatic accumulation patterns in poplar are not dominated by our estimate of tree age, we also examined the accumulation patterns within tree 13 and tree 14 separately. We found similar accumulation slopes as well as epimutation rates (Fig. 4h, see also co-submission [26]).

#### Epimutation spectra have a somatic origin

We examined the somatic epimutation rate estimates from the complete tree analysis. At the genome-wide scale, we found that the 5mC gain and loss rates in context CG are 1.7·10^−6^ and 5.8·10^−6^ per site per haploid genome per year, respectively, and 3.3·10^−7^ and 4.1·10^−6^ in context CHG. Interestingly, these *per-year* CG epimutation rates are only about two orders of magnitude lower than the *per-generation* rates in *A. thaliana* MA lines. Assuming an average generation time of about 15 to 150 years in poplar [47], its expected per-generation CG epimutation rate would be between ∼10^−5^ and ∼10^−4^, which is within the same order of magnitude to that of *A. thaliana* (∼10^−4^) (Fig. 3a). This close similarity is remarkable given that poplar is about ∼ 100 times larger and its life cycle ∼ 1000 times longer than that of *A. thaliana*. Similar insights were reached in a recent comparison of the per-generation nucleotide mutation rates between Oak (*Quercus rubur*) and *A. thaliana* [45], which were also found to be remarkably close to each other. Taken together, these findings support the emerging hypothesis that meristematic cells of long-lived perennials undergo fewer cell divisions per unit time than annuals, so that the cumulative life-time number of cell divisions is similar [46]. This hypothesis should be tested more directly using cell count assays.

To assess whether the accumulation dynamics of somatic epimutations in poplar differs between genomic features, we examined in more detail the estimated rates and spectra for exons, promoters, and TEs (Fig. 3b). Focusing on context CG, we found considerable rate differences. The gain rates for exons, promoters, and TEs were 2.4·10^−6^,1.1·10^−6^, and 7.5·10^−7^ per site per haploid genome per year, respectively, and the loss rates were 2·10^−5^,8·10^−6^, and 2.8·10^−7^. Intriguingly, the rank order of these rates was similar to what we had observed for germline epimutations in *A. thaliana*, with exons showing the highest combined rates, followed by promoters and then TEs (Fig. 3b). These findings indicate that the epimutation spectrum is deeply conserved across angiosperms and that it is mainly shaped during somatic development, rather than being a byproduct of selective reinforcement of DNA methylation in the germline or early zygote. Identifying *cis*- and *trans*-determinants that affect local epimutation rates seems to be an important next challenge [11].

### Analysis spontaneous epimutations in asexually derived dandelion MA lines

**Fig. 5:**
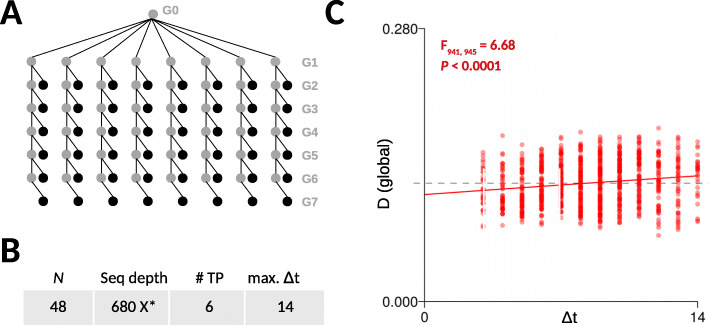
Analysis of CG epimutations in apomictic dandelion. **a** Using single-seed descent from a single apomictic triploid founder genotype, 8 replicated lineages were propagated for 6 generations. DNA methylation measurements were obtained using epigenotyping by sequencing (epiGBS). **b** Overview of the data: *N* is the total number of sequenced samples; Seq depth is the average sequence depths of the samples; # TP is the number of unique time-points that are sampled; max. *Δ*t is the maximum divergence time (in generations) between samples. *Note: the calculation of average read coverage was based only on interrogated cytosines as epiGBS does not yield any genome-wide data. **c** Model fits to the CG divergence data. Highly significant increases in 5mC divergence (*D*) over generation time (*Δt*) were detected in all sequence contexts, despite the relatively large variation in 5mC divergence patterns (see text)

Our analysis of *A. thaliana* and poplar revealed strong similarities in epimutation rates and spectra. To facilitate further inter-specific comparisons, particulary across different mating systems, we generated novel MA lines in an asexual dandelion (*Taraxacum officinale*) genotype (AS34) [48] (Fig. 5a). Apomictic dandelions are triploid and produce asexually via clonal seeds in a process that involves unreduced egg cell formation (diplospory), parthenogenic embryo development, and autonomous endosperm formation, resulting in genetically identical offspring [49]. Using single-seed descent from a single apomictic triploid founder genotype, 8 replicated lineages were propagated for 6 generations, and 5mC measurements were obtained from each generation (Fig. 5a).

The total dataset was relatively large, with 48 sequenced samples and a maximum divergence time of 14 generations (Fig. 5b). 5mC measurements were obtained using epigenotyping-by-sequencing (epiGBS) [32] (see the “Materials and data pre-processing” section). Since there is currently no published dandelion reference assembly, local assemblies were generated de novo from the epiGBS short reads and served as basis for cytosine methylation calling [32]. With this approach, ∼ 24000 measured cytosines were shared between any two sample pairs on average and were used to calculate pair-wise CG methylation divergence *D*.

Plotting *D* against divergence time (*Δt*) revealed considerable measurement variation across samples (Fig. 5c). This large variation could have several possible sources: First, methylation state calling was based on local assemblies rather than on reference-based alignments. Second, epiheterozygotes in this triploid genotype could not be effectively distinguished on the basis of the observed methylation levels, which introduce uncertainties in the calculation *D*. Third, early implementations of the epiGBS protocol could not distinguish PCR duplicates, a problem that has since been solved [50].

Despite these limitations, application of *AlphaBeta* to the CG divergence data revealed strong statistical evidence for epimutation accumulation over time (*F*_941,945_=6.68, *p*< 0.0001). Consistent with *A. thaliana* and poplar, a neutral epimutation model (*ABneutral*) provided the best fit to the data. Based on these model fits, we estimate the global CG gain rate and loss rate at 6.9·10^−4^ and 1.4·10^−3^ per CG site per haploid genome per generation, respectively (Fig. 3). We note that these “per-haploid” rate estimates are slightly biased upward, since we applied *AlphaBeta*’s diploid models to data from a triploid species, but this model mis-specification should have little impact in the analysis of asexually reproducing systems in which genetic segregation is absent. Keeping this caveat in mind, our results show that the dandelion per-generation CG epimutation rates are close to those obtained in *A. thaliana* and poplar (Fig. 3a), and at least within the same order of magnitude. This finding reinforces the notion that epimutational process are largely conserved across angiosperms, which is probably a direct consequence of the fact that the DNA methylation maintenance machinery is itself highly conserved [5, 51]. Moreover, our findings in dandelion lend further support to the hypothesis that sexual reproduction has no major impact on the formation and inheritance of spontaneous epimutations. Future studies should test this hypothesis more directly by studying the epimutation landscape of a fixed genotype that has been propagated in parallel both sexually and asexually.

## Discussion

Accurate estimates of the rate and spectrum of spontaneous epimutations are essential for understanding how DNA methylation diversity arises in the context of plant evolution, development, and aging. Here, we presented *AlphaBeta*, a computational method for obtaining such estimates from pedigree-based high-throughput DNA methylation data. Our method requires that the topology of the pedigree is known. This requirement is typically met in the experimental construction of mutation accumulation lines (MA lines) that are derived through sexual or clonal reproduction. However, we demonstrated that *AlphaBeta* can also be used to study somatic epimutations in long-lived perennials, such as trees, using leaf methylomes and coring data as input. In this case, our method treats the tree branching structure as an intra-organismal phylogeny of somatic lineages and uses information about the epimutational history of each branch.

To demonstrate the versatility of our method, we applied *AlphaBeta* to very diverse plant systems, including multi-generational DNA methylation data from selfing- and asexually derived MA lines of *A. thaliana* and dandelion, as well as intra-generational DNA methylation data of a poplar tree. Our analysis led to several novel insights about epimutational processes in plants. One of the most striking findings was the close similarity in the epimutation landscapes between these very different systems. Close similarities were observed in the per-generation CG epimutation rates between *A. thaliana*, dandelion, and poplar both at the genome-wide as well as at the subgenomic scale. Any detected rate differences between these different systems were all within one order of a magnitude of each other, and as such practically indistinguishable from experimental sources of variation. As a reference, variation in epimutation rate estimates across different *A. thaliana* mutation accumulation experiments vary up to 75% of an order of a magnitude. Clearly, larger sample sizes are needed along with controlled experimentally comparisons to be able to identify potential biological causes underlying subtle epimutation rate differences between species, mating systems, genotypes, or environmental treatments. Furthermore, the close similarity between sexual and asexual (or somatic) systems reported here provide indirect evidence that transgenerationally heritable epimutations originate mainly during mitotic rather than during meiotic cell divisions in plants.

Our application of *AlphaBeta* to poplar also provided the first proof-of-principle demonstration that leaf methylome data, in combination with our statistical models, can be employed as a molecular clock to age-date trees or sectors of trees. Analytically, this is similar to inferring the branch lengths of the underlying pedigree (or phylogeny). With sufficiently large sample sizes, it should be possible to achieve this with relatively high accuracy and extend this inference to the entire tree structure. The comparatively high rates of somatic and germline epimutations are instrumental in this as they provide increased temporal resolution over classical DNA sequence approaches, which rely on rare de novo nucleotide mutations. Our methodological approach should be applicable, more generally, to any perennial or long-lived species. We are currently extending the *AlphaBeta* tool set to facilitate such analyses.

Analytically, *AlphaBeta* is not restricted to the analysis of plant data. The method could also be used to study epimutational processes in tumor clones based on animal single-cell WGBS data. Such datasets are rapidly emerging [52]. In this context, *AlphaBeta* could be instrumental in the inference of clonal phylogenies and help calibrate them temporally. Such efforts may complement current pseudotemporal ordering (or trajectory inference) methods and lineage tracing strategies in single-cell methylation data [53, 54].

The implementation of *AlphaBeta* is relatively straight-forward. The starting point of the method are methylation state calls for each cytosine. These can be obtained from any methylation calling pipeline. In the data applications presented here, we used *AlphaBeta* in conjunction with *MethylStar* [42], which is an efficient pre-processing pipeline for the analysis of WGBS data and features a HMM-based methylation state caller [55]. Application of this pipeline leads to up a substantial increase in the number of high-confidence cytosine methylation calls for epimutation rate inference compared with more conventional methods. We therefore recommend using *AlphaBeta* in conjunction with *MethylStar*. Software implementing *AlphaBeta* is available as a Bioconductor R package at https://bioconductor.org/packages/release/bioc/html/AlphaBeta.html.

## Materials and data pre-processing

### *A. thaliana*MA lines data

#### Plant material

For MA3, seeds were planted and grown in 16-h day lengths and samples were harvested from young above ground tissue. Tissue was flash frozen in liquid nitrogen and DNA was isolated using a Qiagen Plant DNeasy kit (Qiagen, Valencia, CA, USA) according to the manufacturer’s instructions. For MA1_1 and MA1_3, a detailed description of growth conditions and plant material can be found in the original publications [12, 13].

#### Sequencing and data processing

For MA3, MethylC-seq libraries were prepared according to the protocol described in Urich et al. [56]. Libraries were sequenced to 150 bp per read at the Georgia Genomics & Bioinformatics Core (GGBC) on a NextSeq500 platform (Illumina). Average sequencing depth was 20.8X among samples (Additional file 1: Table S1). For MA1_1 and MA1_3, FASTQ files (*.fastq) were downloaded from https://www.ncbi.nlm.nih.gov/geo/query/acc.cgi?acc=GSE64463. All data processing and methylation state calling was performed using the *MethylStar* pipeline [42]. Summary statistic for each sample can be found in Additional file 1: Table S1. All sequences have been submitted to the GEO repository with the following GEO accession number GSE153055.

### Poplar data

#### Tree coring

The tree used in this study was located at Hood River Ranger District [Horse Thief Meadows area], Mt. Hood National Forest, 0.6 mi south of Nottingham Campground off OR-35 at unmarked parking area, $\phantom {\dot {i}\!}500^{\prime }$ west of East Fork Trail nbr. 650 across river, ca. 45.355313, -121.574284. Tree cores were originally collected from the main stem and five branches in April 2015 at breast height (∼1.5 m) for standing tree age using a stainless-steel increment borer (5 mm in diameter and up to 28 cm in length). Cores were mounted on grooved wood trim, dried at room temperature, sanded, and stained with 1% phloroglucinol following the manufacturer’s instructions (https://www.forestry-suppliers.com/Documents/1568_msds.pdf).

Annual growth rings were counted to estimate age. For cores for which accurate estimates could not be made from the 2015 collection, additional collections were made in spring 2016. However, due to difficulty in collecting by climbing, many of the cores did not reach the center of the stem or branches (pith) and/or the samples displayed heart rot. Combined with the difficulty in demarcating rings in porous woods such as poplar *Populus*, accurate measures of tree age or branch age were challenging.

#### Sequencing and data processing

A single MethylC-seq library was created for each branch from leaf tissue. Libraries were prepared according to the protocol described in Urich et al. [56]. Libraries were sequenced to 150 bp per read at the Georgia Genomics & Bioinformatics Core (GGBC) on a NextSeq500 platform (Illumina). Average sequencing depth was 41.1x among samples. MethylC-seq reads were aligned using Methylpy v1.3.2 [57]. Alignment was to the new Stettler14 assembly of *P. trichocarpa*, as described in [26]. Starting from the BAM files (*.bam), the *MethylStar* pipeline [42] was used for further data processing and methylation state calling. All sequences have been deposited in SRA (see [26]).

### Dandelion MA lines data

#### Plant material

Starting from a single founder individual, eight replicate lineages of the apomictic common dandelion (*Taraxacum officinale*) genotype AS34 [48] were grown for six generations via single-seed descent under common greenhouse conditions. Apomictic dandelions are triploid and produce asexually via clonal seeds in a process that involves unreduced egg cell formation (diplospory), parthenogenic embryo development, and autonomous endosperm formation, resulting in genetically identical offspring [49]. Seeds were collected from each of the 48 plants in the six-generation experiment and stored under controlled conditions (15 ^∘^C and 30% RH). After the 6th generation, from each plant in the pedigree, a single offspring individual was grown in a fully randomized experiment under common greenhouse conditions. Leaf tissue from a standardized leaf was collected after 5 weeks, flash frozen in liquid nitrogen, and stored at − 80 ^∘^C until processing.

#### Sequencing and data processing

DNA was isolated using the Macherey-Nagel Nucleospin Plant II kit (cell lysis buffer PL1). DNA was digested with the PstI restriction enzyme and epiGBS sequencing libraries were prepared as described elsewhere [32]. Based on genotyping-by-sequencing [58], epiGBS is a multiplex reduced representation bisulphite sequencing (RRBS) approach with an analysis pipeline that allows for local reference construction from bisulphite reads, which makes the method applicable to species for which a reference genome is lacking [32]. PstI is a commonly used restriction enzyme for genotyping-by-sequencing; however, its activity is sensitive to CHG methylation in CTGCAG recognition sequence. This makes the enzyme better at unbiased quantification of CG methylation than of CHG methylation [32]. After quantification of the sequencing libraries using a multiplexed Illumina MiSeq Nano run, samples were re-pooled to achieve equal representation in subsequent epiGBS library sequencing. The experimental samples were sequenced on two Illumina HiSeq 2500 lanes (125 cycles paired-end) as part of a larger epiGBS experiment which consisted of a total of 178 samples that were randomized over the two lanes. Because of inadequate germination or due to low sequencing output (library failure), four of the 48 samples were not included in the downstream analysis. All sequences have been deposited in SRA under Bioproject: PRJNA608438. The biosamples include SAMN14266774 to 778, SAMN14266797 to 802, SAMN14266821 to 826, SAMN14266845 to 850, SAMN14266869 to 872, SAMN14266874, SAMN14266893 to 894, SAMN14266896 to 897, SAMN14266916 to 921, and SAMN14266940 to 945. These 44 samples have been submitted as part of a bigger experiment of 178 samples total.

#### DNA methylation analysis

Sequencing reads were demultiplexed (based on custom barcodes) and mapped against a dandelion pseudo-reference sequence that was generated de novo from PstI-based epiGBS [32]. This pseudo-reference contains the local reference of PstI-based epiGBS fragments as inferred from the bisulphite reads. Methylation variant calling was based on SAMtools mpileup and custom python scripts, following a similar approach as described in van Gurp et al. [32]. For downstream analysis, we included only those cytosines that were called in at least 80% of the samples. In addition, cytosine positions that did not pass the filtering criteria for all generations were removed.

To obtain methylation status calls, we implemented a one-tail binomial test as previously described [12]. Multiple testing correction was performed using the Benjamini-Yekutiely method [59], and the false discovery rate (FDR) was controlled at 0.05. All statistical tests for obtaining methylation status calls of the samples were conducted within the SciPy ecosystem.

## Supplementary information

**Additional file 1** **Table S1**. WGBS information for MA pedigrees MA1_1, MA1_3 and MA3.

**Additional file 2** **Table S2**. Epimutation rate estimates and model selection results for pedigree MA1_1.

**Additional file 3** **Table S3**. Epimutation rate estimates and model selection results for pedigree MA1_3.

**Additional file 4** **Table S4**. Epimutation rate estimates and model selection results for pedigree MA3.

**Additional file 5** **Table S5**. Pre-processing of WGBS data using MethylStar increases the number of high-confident cytosines that can be used for epimutation analysis compared with previous pre-processing approaches.

**Additional file 6** **Figure S1**. Developmental origin of somatic epimutations in plants.

**Additional file 7** Review history.

## Data Availability

AlphaBeta [60] is an open source R package licensed under GPL-3. It is freely and openly available from the Github website (https://github.com/jlab-code/AlphaBeta) under GNU General Public License v3.0, and it is part of Bioconductor [61]. Schmitz RJ. AlphaBeta: Computational inference of epimutation rates and spectra from high-throughput DNA methylation data in plants. GSE153055. https://www.ncbi.nlm.nih.gov/geo/query/acc.cgi?acc=GSE153055(2020) [62]. Van Gurp TP, Wagemaker NCAM, Verhoeven KJF. Epimutation accumulation experiment in two Taraxacum officinale apomicts. BioProject PRJNA608438. https://www.ncbi.nlm.nih.gov/sra/?term=PRJNA608438.

## Abbreviations

WGBS: Whole-genome bisulfite sequencing
TEs: Transposable elements
MA lines: Mutation accumulation lines
RRBS: Reduced representation bisulphite sequencing
epiGBS: Epigenotyping by sequencing
5mC: 5-Methyl cytosine
RdDM: RNA-directed DNA methylation pathway
CMT2: CHROMOMETHYLASE 2
HMM: Hidden Markov model
FDR: False discovery rate

## References

[1] Law JA, Jacobsen SE (2010). Establishing, maintaining and modifying DNA methylation patterns in plants and animals. Nat Rev Genet.

[2] Stroud H, Greenberg MVC, Feng S, Bernatavichute YV, Jacobsen SE (2013). Comprehensive analysis of silencing mutants reveals complex regulation of the Arabidopsis methylome. Cell.

[3] Bewick AJ, Hofmeister BT, Powers RA, Mondo SJ, Grigoriev IV, James TY, Stajich JE, Schmitz RJ (2019). Diversity of cytosine methylation across the fungal tree of life. Nat Ecol Evol.

[4] Feng S, Cokus SJ, Zhang X, Chen P-Y, Bostick M, Goll MG, Hetzel J, Jain J, Strauss SH, Halpern ME, Ukomadu C, Sadler KC, Pradhan S, Pellegrini M, Jacobsen SE. Proc Natl Acad Sci USA. 2010; 107(19):8689–94. 10.1073/pnas.1002720107. 10.1073/pnas.1002720107 PMC2889301 20395551

[5] Niederhuth CE, Bewick AJ, Ji L, Alabady MS, Kim KD, Li Q, Rohr NA, Rambani A, Burke JM, Udall JA, Egesi C, Schmutz J, Grimwood J, Jackson SA, Springer NM, Schmitz RJ. Widespread natural variation of DNA methylation within angiosperms. Genome Biol. 2016; 17. 10.1186/s13059-016-1059-0. 10.1186/s13059-016-1059-0 PMC5037628 27671052

[6] Takuno S, Ran J-H, Gaut BS (2016). Evolutionary patterns of genic DNA methylation vary across land plants. Nat Plants.

[7] Zemach A, McDaniel IE, Silva P, Zilberman D (2010). Genome-wide evolutionary analysis of eukaryotic DNA methylation. Science (New York, NY).

[8] Field AE, Robertson NA, Wang T, Havas A, Ideker T, Adams PD (2018). DNA methylation clocks in aging: categories, causes, and consequences. Mol Cell.

[9] Calarco JP, Borges F, Donoghue MTA, Van Ex F, Jullien PE, Lopes T, Gardner R, Berger F, Feijó JA, Becker JD, Martienssen RA (2012). Reprogramming of DNA methylation in pollen guides epigenetic inheritance via small RNA. Cell.

[10] Walker J, Gao H, Zhang J, Aldridge B, Vickers M, Higgins JD, Feng X (2018). Sexual-lineage-specific DNA methylation regulates meiosis in Arabidopsis. Nat Genetics.

[11] Johannes F, Schmitz RJ (2019). Spontaneous epimutations in plants. New Phytologist.

[12] Graaf AVD, Wardenaar R, Neumann DA, Taudt A, Shaw RG, Jansen RC, Schmitz RJ, Colomé-Tatché M, Johannes F (2015). Rate, spectrum, and evolutionary dynamics of spontaneous epimutations. Proc Natl Acad Sci.

[13] Becker C, Hagmann J, Müller J, Koenig D, Stegle O, Borgwardt K, Weigel D (2011). Spontaneous epigenetic variation in the *Arabidopsis thaliana* methylome. Nature.

[14] Schmitz RJ, Schultz MD, Lewsey MG, O’Malley RC, Urich MA, Libiger O, Schork NJ, Ecker JR (2011). Transgenerational epigenetic instability is a source of novel methylation variants. Science (New York, NY).

[15] Ossowski S, Schneeberger K, Lucas-Lledó JI, Warthmann N, Clark RM, Shaw RG, Weigel D, Lynch M (2010). The rate and molecular spectrum of spontaneous mutations in Arabidopsis thaliana. Science (New York, NY).

[16] Weng M-L, Becker C, Hildebrandt J, Neumann M, Rutter MT, Shaw RG, Weigel D, Fenster CB (2019). Fine-grained analysis of spontaneous mutation spectrum and frequency in Arabidopsis thaliana. Genetics.

[17] Vidalis A, živković D, Wardenaar R, Roquis D, Tellier A, Johannes F (2016). Methylome evolution in plants. Genome Biol.

[18] Hofmeister BT, Lee K, Rohr NA, Hall DW, Schmitz RJ (2017). Stable inheritance of DNA methylation allows creation of epigenotype maps and the study of epiallele inheritance patterns in the absence of genetic variation. Genome Biol.

[19] Hagmann J, Becker C, Müller J, Stegle O, Meyer RC, Wang G, Schneeberger K, Fitz J, Altmann T, Bergelson J, Borgwardt K, Weigel D (2015). Century-scale methylome stability in a recently diverged Arabidopsis thaliana lineage. PLoS Genet.

[20] Schmid MW, Heichinger C, Schmid DC, Guthörl D, Gagliardini V, Bruggmann R, Aluri S, Aquino C, Schmid B, Turnbull LA, Grossniklaus U (2018). Contribution of epigenetic variation to adaptation in Arabidopsis. Nat Commun.

[21] Taudt A, Colomé-Tatché M, Johannes F (2016). Genetic sources of population epigenomic variation. Nat Rev Genet.

[22] Jiang C, Mithani A, Belfield EJ, Mott R, Hurst LD, Harberd NP (2014). Environmentally responsive genome-wide accumulation of de novo Arabidopsis thaliana mutations and epimutations. Genome Res.

[23] Ganguly DR, Crisp PA, Eichten SR, Pogson BJ (2017). The Arabidopsis DNA methylome is stable under transgenerational drought stress. Plant Phys.

[24] Zheng X, Chen L, Xia H, Wei H, Lou Q, Li M, Li T, Luo L (2017). Transgenerational epimutations induced by multi-generation drought imposition mediate rice plant’s adaptation to drought condition. Sci Rep.

[25] Lanfear R (2018). Do plants have a segregated germline?. PLOS Biol.

[26] Hofmeister BT, et al.A genome assembly and the somatic genetic and epigenetic mutation rate in a wild long-lived perennial Populus trichocarpa. Genome Biol. 2020. 10.1186/s13059-020-02162-5. 10.1186/s13059-020-02162-5 PMC7539514 33023654

[27] Horvath R, Laenen B, Takuno S, Slotte T. Single-cell expression noise and gene-body methylation in Arabidopsis thaliana. Heredity. 2019; 1. 10.1038/s41437-018-0181-z. 10.1038/s41437-018-0181-z PMC6781109 30651589

[28] Secco D, Wang C, Shou H, Schultz MD, Chiarenza S, Nussaume L, Ecker JR, Whelan J, Lister R. Stress induced gene expression drives transient DNA methylation changes at adjacent repetitive elements. eLife. 2015; 4. 10.7554/eLife.09343. 10.7554/eLife.09343 PMC4534844 26196146

[29] Cokus SJ, Feng S, Zhang X, Chen Z, Merriman B, Haudenschild CD, Pradhan S, Nelson SF, Pellegrini M, Jacobsen SE (2008). Shotgun bisulphite sequencing of the *Arabidopsis* genome reveals DNA methylation patterning. Nature.

[30] Lister R, O’Malley RC, Tonti-Filippini J, Gregory BD, Berry CC, Millar AH, Ecker JR (2008). Highly integrated single-base resolution maps of the epigenome in Arabidopsis. Cell.

[31] Meissner A, Gnirke A, Bell GW, Ramsahoye B, Lander ES, Jaenisch R (2005). Reduced representation bisulfite sequencing for comparative high-resolution DNA methylation analysis. Nucleic Acids Res.

[32] van Gurp TP, Wagemaker NCAM, Wouters B, Vergeer P, Ouborg JNJ, Verhoeven KJF (2016). epiGBS: reference-free reduced representation bisulfite sequencing. Nat Methods.

[33] Colomé-Tatché M, Johannes F (2016). Signatures of Dobzhansky–Muller incompatibilities in the genomes of eecombinant inbred lines. Genetics.

[34] Broman KW (2012). Genotype probabilities at intermediate generations in the construction of recombinant Inbred Lines. Genetics.

[35] Johannes F, Colomé-Tatché M (2011). Quantitative epigenetics through epigenomic perturbation of isogenic lines. Genetics.

[36] Bartlett MS, Haldane JBS (1935). The theory of inbreeding with forced heterozygosis. J Genet.

[37] Ronald Aylmer Fisher (1949). The theory of inbreeding.

[38] Kawashima T, Berger F (2014). Epigenetic reprogramming in plant sexual reproduction. Nat Rev Genet.

[39] Gehring M. Epigenetic dynamics during flowering plant reproduction: evidence for reprogramming?New Phytol. 10.1111/nph.15856. 10.1111/nph.15856 PMC6711810 31002174

[40] Teixeira FK, Heredia F, Sarazin A, Roudier F, Boccara M, Ciaudo C, Cruaud C, Poulain J, Berdasco M, Fraga MF, Voinnet O, Wincker P, Esteller M, Colot V (2009). A role for RNAi in the selective correction of DNA methylation defects. Science.

[41] Gouil Q, Baulcombe DC (2016). DNA methylation signatures of the plant chromomethyltransferases. PLOS Genet.

[42] Shahryary Y, Hazarika RR, Johannes F (2020). Methylstar: a fast and robust pre-processing pipeline for bulk or single-cell whole-genome bisulfite sequencing data. BMC Genomics.

[43] Wang L, Ji Y, Hu Y, Hu H, Jia X, Jiang M, Zhang X, Zhao L, Zhang Y, Jia Y, Qin C, Yu L, Huang J, Yang S, Hurst LD, Tian D (2019). The architecture of intra-organism mutation rate variation in plants. PLOS Biol.

[44] Hanlon VCT, Otto SP, Aitken SN. Somatic mutations substantially increase the per-generation mutation rate in the conifer Picea sitchensis. Evol Lett. 10.1002/evl3.121. 10.1002/evl3.121 PMC6675141 31388445

[45] Schmid-Siegert E, Sarkar N, Iseli C, Calderon S, Gouhier-Darimont C, Chrast J, Cattaneo P, Schütz F, Farinelli L, Pagni M, Schneider M, Voumard J, Jaboyedoff M, Fankhauser C, Hardtke CS, Keller L, Pannell JR, Reymond A, Robinson-Rechavi M, Xenarios I, Reymond P (2017). Low number of fixed somatic mutations in a long-lived oak tree. Nat Plants.

[46] Orr AJ, Padovan A, Kainer D, Külheim C, Bromham L, Bustos-Segura C, Foley W, Haff T, Hsieh J-F, Morales-Suarez A, Cartwright RA, Lanfear R. A phylogenomic approach reveals a low somatic mutation rate in a long-lived plant. bioRxiv. 2019:727982. 10.1101/727982. 10.1098/rspb.2019.2364 PMC7126060 32156194

[47] Ingvarsson PK (2008). Multilocus patterns of nucleotide polymorphism and the demographic history of Populus tremula. Genetics.

[48] Verhoeven KJF, Van Dijk PJ, Biere A (2010). Changes in genomic methylation patterns during the formation of triploid asexual dandelion lineages. Mol Ecol.

[49] Koltunow A (1993). Apomixis: embryo sacs and embryos formed without meiosis or fertilization in ovules,. Plant Cell.

[50] Moorsel S. J. v., Schmid MW, Wagemaker NCAM, Gurp T. v., Schmid B, Vergeer P. Evidence for rapid evolution in a grassland biodiversity experiment. bioRxiv. 2018:262303. 10.1101/262303. 10.1111/mec.15191 31336411

[51] Bewick AJ, Niederhuth CE, Ji L, Rohr NA, Griffin PT, Leebens-Mack J, Schmitz RJ (2017). The evolution of CHROMOMETHYLASES and gene body DNA methylation in plants. Genome Biol.

[52] Gaiti F, Chaligne R, Gu H, Brand RM, Kothen-Hill S, Schulman RC, Grigorev K, Risso D, Kim K-T, Pastore A, Huang KY, Alonso A, Sheridan C, Omans ND, Biederstedt E, Clement K, Wang L, Felsenfeld JA, Bhavsar EB, Aryee MJ, Allan JN, Furman R, Gnirke A, Wu CJ, Meissner A, Landau DA. Epigenetic evolution and lineage histories of chronic lymphocytic leukaemia. Nature. 2019; 1. 10.1038/s41586-019-1198-z. 10.1038/s41586-019-1198-z PMC6533116 31092926

[53] Danese A, Richter ML, Fischer DS, Theis FJ, Colomé-Tatché M. EpiScanpy: integrated single-cell epigenomic analysis. bioRxiv. 2019:648097. 10.1101/648097. 10.1038/s41467-021-25131-3 PMC8410937 34471111

[54] Saelens W, Cannoodt R, Todorov H, Saeys Y (2019). A comparison of single-cell trajectory inference methods. Nat Biotechnol.

[55] Taudt A, Roquis D, Vidalis A, Wardenaar R, Johannes F, Colomé-Tatché M (2018). METHimpute: imputation-guided construction of complete methylomes from WGBS data. BMC Genomics.

[56] Urich MA, Nery JR, Lister R, Schmitz RJ, Ecker JR (2015). MethylC-seq library preparation for base-resolution whole-genome bisulfite sequencing. Nat Protocol.

[57] Schultz MD, He Y, Whitaker JW, Hariharan M, Mukamel EA, Leung D, Rajagopal N, Nery JR, Urich MA, Chen H, Lin S, Lin Y, Jung I, Schmitt AD, Selvaraj S, Ren B, Sejnowski TJ, Wang W, Ecker JR (2015). Human body epigenome maps reveal noncanonical DNA methylation variation. Nature.

[58] Elshire RJ, Glaubitz JC, Sun Q, Poland JA, Kawamoto K, Buckler ES, Mitchell SE (2011). A robust, simple genotyping-by-sequencing (GBS) approach for high diversity species. PLOS ONE.

[59] Benjamini Y, Yekutieli D (2001). The control of the false discovery rate in multiple testing under dependency. Ann Stat.

[60] Shahryary Y, Johannes F, Hazarika R. jlab-code/AlphaBeta. 2020. 10.5281/zenodo.3992612.

[61] Shahryary Y, Johannes F, Hazarika R. Bioconductor AlphaBeta Software Package. 2020. 10.18129/B9.bioc.AlphaBeta.

[62] Schmitz RJ. AlphaBeta: Computational inference of epimutation rates and spectra from high-throughput DNA methylation data in plants. GSE153055. 2020. https://urldefense.proofpoint.com/v2/url?u=https-3A__www.ncbi.nlm.nih.gov_geo_ query_acc.cgi-3Facc-3DGSE153055&d=DwIGaQ&c=vh6FgFnduejNhPPD0fl_yRaSfZy8CWbWnIf4XJhSqx8&r=Z3BY_DFGt24T_Oe13xHJ2wIDudwzO_8VrOFSUQlQ_zsz-DGcYuoJS3jWWxMQECLm&m= nMao27rggwqbBJbvu1-d0yavK1ZEszYRhgNn0-mmx8g&s=HsUT2FBGvJLvyqtcALnMlH07FzdJt3Uw2EtloId06B0&e=.

