# Supplementary material for: AlphaBeta: computational inference of epimutation rates and spectra from high-throughput DNA methylation data in plants
Source: Genome Biol. 2020 Oct 6;21:260. doi: 10.1186/s13059-020-02161-6 (PMC7539454; doi:10.1186/s13059-020-02161-6)
Supplement: Supplementary file 1 — Additional file 1 Table S1. WGBS information for MA pedigrees MA1_1, MA1_3 and MA3. [file 13059_2020_2161_MOESM1_ESM.pdf]

# Table S1

| samples | platform | library layout | Mean Coverage(X) |
|---|---|---|---|
| MA1_1_G31_109_r1 | Illumina GAIIx | Paired-end | 10.75 |
| MA1_1_G31_109_r2 | Illumina GAIIx | Paired-end | 7.53 |
| MA1_1_G31_119_r1 | Illumina GAIIx | Paired-end | 9.91 |
| MA1_1_G31_119_r2 | Illumina GAIIx | Paired-end | 10.5 |
| MA1_1_G31_29_r1 | Illumina GAIIx | Paired-end | 7.91 |
| MA1_1_G31_29_r2 | Illumina GAIIx | Paired-end | 13.53 |
| MA1_1_G31_29_r3 | Illumina GAIIx | Paired-end | 7.86 |
| MA1_1_G31_39_r1 | Illumina GAIIx | Paired-end | 12.29 |
| MA1_1_G31_39_r2 | Illumina GAIIx | Paired-end | 6.98 |
| MA1_1_G31_49_r1 | Illumina GAIIx | Paired-end | 8.71 |
| MA1_1_G31_49_r2 | Illumina GAIIx | Paired-end | 11.26 |
| MA1_1_G31_59_r1 | Illumina GAIIx | Paired-end | 11.05 |
| MA1_1_G31_59_r2 | Illumina GAIIx | Paired-end | 7.07 |
| MA1_1_G31_79_r1 | Illumina GAIIx | Paired-end | 11.28 |
| MA1_1_G31_79_r2 | Illumina GAIIx | Paired-end | 13.77 |
| MA1_1_G31_89_r1 | Illumina GAIIx | Paired-end | 9.28 |
| MA1_1_G31_89_r2 | Illumina GAIIx | Paired-end | 7.94 |
| MA1_1_G31_99_r1 | Illumina GAIIx | Paired-end | 10.83 |
| MA1_1_G31_99_r2 | Illumina GAIIx | Paired-end | 7.18 |
| MA1_1_G31_99_r3 | Illumina GAIIx | Paired-end | 12.96 |
| MA1_1_G32_39_r1 | Illumina GAIIx | Paired-end | 9.81 |
| MA1_1_G32_39_r2 | Illumina GAIIx | Paired-end | 9.43 |
| MA1_1_G32_49_r1 | Illumina GAIIx | Paired-end | 8.65 |
| MA1_1_G32_49_r2 | Illumina GAIIx | Paired-end | 6.6 |
| MA1_1_G3_26_r1 | Illumina GAIIx | Paired-end | 7.99 |
| MA1_1_G3_87_r1 | Illumina GAIIx | Paired-end | 10.97 |
| MA1_1_G3_87_r2 | Illumina GAIIx | Paired-end | 7.66 |
| MA1_3_G18_12_r1 | Illumina NextSeq 500 | Single-end | 5.86 |
| MA1_3_G19_12_r1 | Illumina NextSeq 500 | Single-end | 6.25 |
| MA1_3_G20_12_r1 | Illumina NextSeq 500 | Single-end | 6.56 |
| MA1_3_G21_12_r1 | Illumina NextSeq 500 | Single-end | 8.55 |
| MA1_3_G25_12_r1 | Illumina NextSeq 500 | Single-end | 6.46 |
| MA1_3_G26_12_r1 | Illumina NextSeq 500 | Single-end | 6.75 |
| MA1_3_G28_12_r1 | Illumina NextSeq 500 | Single-end | 8.18 |
| MA1_3_G29_12_r1 | Illumina NextSeq 500 | Single-end | 6.67 |
| MA1_3_G30_12_r1 | Illumina NextSeq 500 | Single-end | 7.2 |
| MA3_G0 | Illumina NextSeq 500 | Single-end | 9.44 |
| MA3_G11_L2 | Illumina NextSeq 500 | Single-end | 10.31 |
| MA3_G11_L8 | Illumina NextSeq 500 | Single-end | 23.52 |
| MA3_G1_L2 | Illumina NextSeq 500 | Single-end | 8.85 |
| MA3_G1_L8 | Illumina NextSeq 500 | Single-end | 8.47 |
| MA3_G2_L2 | Illumina NextSeq 500 | Single-end | 9.91 |
| MA3_G2_L8 | Illumina NextSeq 500 | Single-end | 9.36 |
| MA3_G4_L2 | Illumina NextSeq 500 | Single-end | 9.8 |
| MA3_G4_L8 | Illumina NextSeq 500 | Single-end | 9.38 |
| MA3_G5_L2 | Illumina NextSeq 500 | Single-end | 8.82 |
| MA3_G5_L8 | Illumina NextSeq 500 | Single-end | 8.98 |
| MA3_G8_L2 | Illumina NextSeq 500 | Single-end | 8.97 |
| MA3_G8_L8 | Illumina NextSeq 500 | Single-end | 9.43 |

**Table S1**: WGBS information for MA pedigrees MA1_1, MA1_3 and MA3**.**
