# Supplementary material for: AlphaBeta: computational inference of epimutation rates and spectra from high-throughput DNA methylation data in plants
Source: Genome Biol. 2020 Oct 6;21:260. doi: 10.1186/s13059-020-02161-6 (PMC7539454; doi:10.1186/s13059-020-02161-6)
Supplement: Supplementary file 2 — Additional file 2 Table S2. Epimutation rate estimates and model selection results for pedigree MA1_1. [file 13059_2020_2161_MOESM2_ESM.pdf]

Table S2

A. thaliana (MA1_1)

context	annotation	alpha	beta	beta/alpha	FM	RM	F-value	df RM	df FM	P--value
CG	global	8.605897E-05	0.0002497981	2.903	ABneutral	Abnull	461.7119	350	346	2.70488E-137
CG	exon	0.0003329146	0.0008854249	2.660	ABneutral	Abnull	506.9881	350	346	2.99272E-143
CG	promoter	4.390624E-05	0.0003396206	7.735	ABneutral	Abnull	574.9184	350	346	2.19508E-151
CG	TE	2.777663E-05	7.500164E-06	0.270	ABneutral	Abnull	122.3618	350	346	6.001881E-65
CG	global				ABselectUU	Abneutral	0.4806	347	346	0.4885955
CG	exon				ABselectUU	Abneutral	0.0453	347	346	0.8316087
CG	promoter				ABselectUU	Abneutral	1.8765	347	346	0.1716251
CG	TE				ABselectUU	Abneutral	0.7210	347	346	0.3963924
CG	global				ABselectMM	Abneutral	0.4731	347	346	0.4920452
CG	exon				ABselectMM	Abneutral	0.1176	347	346	0.7318225
CG	promoter				ABselectMM	Abneutral	0.4049	347	346	0.5249724
CG	TE				ABselectMM	Abneutral	0.1476	347	346	0.7011182
CHG	global	3.533486E-06	5.84628E-05	16.545	ABneutral	Abnull	16.4449	350	346	2.399368E-12
CHG	exon	1.537786E-06	0.0002547714	165.674	ABneutral	Abnull	20.6207	350	346	2.958294E-15
CHG	promoter	2.408792E-06	0.0001227248	50.949	ABneutral	Abnull	19.3769	350	346	2.124209E-14
CHG	TE	2.124037E-05	3.025496E-05	1.424	ABneutral	Abnull	9.4822	350	346	2.768493E-07
CHG	global				ABselectUU	Abneutral	0.0000	347	346	1
CHG	exon				ABselectUU	Abneutral	0.0164	347	346	0.8980838
CHG	promoter				ABselectUU	Abneutral	0.0000	347	346	1
CHG	TE				ABselectUU	Abneutral	0.0000	347	346	1
CHG	global				ABselectMM	Abneutral	0.0000	347	346	1
CHG	exon				ABselectMM	Abneutral	0.0228	347	346	8.80E-01
CHG	promoter				ABselectMM	Abneutral	0.0000	347	346	1
CHG	TE				ABselectMM	Abneutral	0.0000	347	346	1
CHH	global	1.905105E-06	0.0001614412	84.741	ABneutral	Abnull	11.6186	350	346	7.294017E-09
CHH	exon	1.09903E-06	0.0006017335	547.513	ABneutral	Abnull	18.1246	350	346	1.577799E-13
CHH	promoter	1.349004E-06	0.0002622809	194.426	ABneutral	Abnull	19.3769	350	346	2.124209E-14
CHH	TE	5.54414E-06	6.181445E-05	11.150	ABneutral	Abnull	9.4822	350	346	2.768493E-07
CHH	global				ABselectUU	Abneutral	0.9755	347	346	0.3240002
CHH	exon				ABselectUU	Abneutral	0.0000	347	346	1
CHH	promoter				ABselectUU	Abneutral	0.0000	347	346	1
CHH	TE				ABselectUU	Abneutral	0.0000	347	346	1
CHH	global				ABselectMM	Abneutral	0.0000	347	346	1
CHH	exon				ABselectMM	Abneutral	0.0000	347	346	1
CHH	promoter				ABselectMM	Abneutral	0.0000	347	346	1
CHH	TE				ABselectMM	Abneutral	0.0000	347	346	1

FM = Full model
RM = Reduced model
df = degrees of freedom
Best performing model

Table S2: Epimutation rate estimates and model selection results for pedigree MA1_1
