# Supplementary material for: AlphaBeta: computational inference of epimutation rates and spectra from high-throughput DNA methylation data in plants
Source: Genome Biol. 2020 Oct 6;21:260. doi: 10.1186/s13059-020-02161-6 (PMC7539454; doi:10.1186/s13059-020-02161-6)
Supplement: Supplementary file 3 — Additional file 3 Table S3. Epimutation rate estimates and model selection results for pedigree MA1_3. [file 13059_2020_2161_MOESM3_ESM.pdf]

Table S3

A. thaliana (MA1_3)

context	annotation	alpha	beta	beta/alpha	FM	RM	F-value	df RM	df FM	P--value
CG	global	0.0001411325	0.0006480785	4.592	ABneutral	Abnull	26.0964	35	31	1.545386E-09
CG	exon	0.0004417924	0.001566664	3.546	ABneutral	Abnull	36.7694	35	31	2.352825E-11
CG	promoter	8.473369E-05	0.0009098872	10.738	ABneutral	Abnull	22.7134	35	31	7.66475E-09
CG	TE	0.0002361935	0.0001108124	0.469	ABneutral	Abnull	7.9028	35	31	0.0001635841
CG	global				ABselectUU	Abneutral	0.6312	32	31	0.4329535
CG	exon				ABselectUU	Abneutral	0.1957	32	31	0.6612462
CG	promoter				ABselectUU	Abneutral	0.4402	32	31	0.5119528
CG	TE				ABselectUU	Abneutral	0.0135	32	31	0.9084139
CG	global				ABselectMM	Abneutral	0.6343	32	31	0.4318425
CG	exon				ABselectMM	Abneutral	0.2816	32	31	0.5994397
CG	promoter				ABselectMM	Abneutral	0.2889	32	31	0.5947381
CG	TE				ABselectMM	Abneutral	0.0163	32	31	0.8993372
CHG	global	NA	NA	NA	ABneutral	Abnull	0.6354	35	31	0.6410907
CHG	exon	NA	NA	NA	ABneutral	Abnull	0.4926	35	31	0.7411286
CHG	promoter	NA	NA	NA	ABneutral	Abnull	0.7356	35	31	0.5747893
CHG	TE	NA	NA	NA	ABneutral	Abnull	0.3041	35	31	0.8729979
CHG	global				ABselectUU	Abneutral	0.0271	32	31	0.8703212
CHG	exon				ABselectUU	Abneutral	0.0000	32	31	1
CHG	promoter				ABselectUU	Abneutral	0.1223	32	31	0.7289703
CHG	TE				ABselectUU	Abneutral	0.0759	32	31	0.7847896
CHG	global				ABselectMM	Abneutral	0.0312	32	31	0.8609342
CHG	exon				ABselectMM	Abneutral	0.0000	32	31	1.00E+00
CHG	promoter				ABselectMM	Abneutral	0.1063	32	31	0.7466025
CHG	TE				ABselectMM	Abneutral	0.0779	32	31	0.7820306
CHH	global	NA	NA	NA	ABneutral	Abnull	0.6695	35	31	0.6180636
CHH	exon	NA	NA	NA	ABneutral	Abnull	0.5049	35	31	0.732369
CHH	promoter	NA	NA	NA	ABneutral	Abnull	1.2939	35	31	0.2938961
CHH	TE	NA	NA	NA	ABneutral	Abnull	0.3691	35	31	0.8287454
CHH	global				ABselectUU	Abneutral	0.0018	32	31	0.9661093
CHH	exon				ABselectUU	Abneutral	0.0000	32	31	1
CHH	promoter				ABselectUU	Abneutral	0.0016	32	31	0.9687817
CHH	TE				ABselectUU	Abneutral	0.7337	32	31	0.3982717
CHH	global				ABselectMM	Abneutral	0.0079	32	31	0.9296158
CHH	exon				ABselectMM	Abneutral	0.0000	32	31	1
CHH	promoter				ABselectMM	Abneutral	0.0003	32	31	0.9871071
CHH	TE				ABselectMM	Abneutral	0.0325	32	31	0.8581938

FM = Full model
RM = Reduced model
df = degrees of freedom
Best performing model

Table S3: Epimutation rate estimates and model selection results for pedigree MA1_3
