# Supplementary material for: AlphaBeta: computational inference of epimutation rates and spectra from high-throughput DNA methylation data in plants
Source: Genome Biol. 2020 Oct 6;21:260. doi: 10.1186/s13059-020-02161-6 (PMC7539454; doi:10.1186/s13059-020-02161-6)
Supplement: Supplementary file 4 — Additional file 4 Table S4. Epimutation rate estimates and model selection results for pedigree MA3. [file 13059_2020_2161_MOESM4_ESM.pdf]

Table S4

A. thaliana (MA3)

context	annotation	alpha	beta	beta/alpha	FM	RM	F-value	df RM	df FM	P--value
CG	global	0.0001942304	0.0008340002	4.294	ABneutral	Abnull	136.2307	65	61	1.103063E-29
CG	exon	0.0005635082	0.001829657	3.247	ABneutral	Abnull	210.1967	65	61	6.177088E-35
CG	promoter	0.0001128601	0.001141507	10.114	ABneutral	Abnull	108.4535	65	61	5.190251E-27
CG	TE	NA	NA	NA	ABneutral	Abnull	1.0608	65	61	0.383711
CG	global				ABselectUU	Abneutral	0.0000	62	61	1
CG	exon				ABselectUU	Abneutral	0.0000	62	61	1
CG	promoter				ABselectUU	Abneutral	0.1053	62	61	0.7467267
CG	TE				ABselectUU	Abneutral	0.0000	62	61	1
CG	global				ABselectMM	Abneutral	0.0001	62	61	0.9934013
CG	exon				ABselectMM	Abneutral	0.0000	62	61	1
CG	promoter				ABselectMM	Abneutral	0.0000	62	61	1
CG	TE				ABselectMM	Abneutral	0.1116	62	61	0.739439
CHG	global	NA	NA	NA	ABneutral	Abnull	2.0182	65	61	0.1030624
CHG	exon	NA	NA	NA	ABneutral	Abnull	0.0141	65	61	0.9995992
CHG	promoter	1.783862E-06	0.0001055335	59.160	ABneutral	Abnull	5.0076	65	61	0.001479819
CHG	TE	5.517707E-05	0.0001307905	2.370	ABneutral	Abnull	5.7078	65	61	0.0005720866
CHG	global				ABselectUU	Abneutral	0.9363	62	61	0.3370451
CHG	exon				ABselectUU	Abneutral	0.0000	62	61	1
CHG	promoter				ABselectUU	Abneutral	0.0707	62	61	0.7912417
CHG	TE				ABselectUU	Abneutral	0.5221	62	61	0.4727021
CHG	global				ABselectMM	Abneutral	0.8975	62	61	0.3471843
CHG	exon				ABselectMM	Abneutral	0.0000	62	61	1.00E+00
CHG	promoter				ABselectMM	Abneutral	0.0000	62	61	1
CHG	TE				ABselectMM	Abneutral	0.8804	62	61	0.3517958
CHH	global	NA	NA	NA	ABneutral	Abnull	1.0187	65	61	0.404864
CHH	exon	NA	NA	NA	ABneutral	Abnull	0.1767	65	61	0.9495845
CHH	promoter	1.71043E-08	4.1274E-06	241.308	ABneutral	Abnull	2.9878	65	61	0.02558529
CHH	TE	NA	NA	NA	ABneutral	Abnull	0.2803	65	61	0.8896153
CHH	global				ABselectUU	Abneutral	0.7073	62	61	0.4036315
CHH	exon				ABselectUU	Abneutral	0.4033	62	61	0.5277759
CHH	promoter				ABselectUU	Abneutral	0.0000	62	61	1
CHH	TE				ABselectUU	Abneutral	0.0000	62	61	1
CHH	global				ABselectMM	Abneutral	1.2129	62	61	0.275082
CHH	exon				ABselectMM	Abneutral	0.2588	62	61	0.6127902
CHH	promoter				ABselectMM	Abneutral	0.0000	62	61	1
CHH	TE				ABselectMM	Abneutral	0.0684	62	61	0.7945567

FM = Full model
RM = Reduced model
df = degrees of freedom
Best performing model

Table S4: Epimutation rate estimates and model selection results for pedigree MA3
