# Supplementary material for: AlphaBeta: computational inference of epimutation rates and spectra from high-throughput DNA methylation data in plants
Source: Genome Biol. 2020 Oct 6;21:260. doi: 10.1186/s13059-020-02161-6 (PMC7539454; doi:10.1186/s13059-020-02161-6)
Supplement: Supplementary file 5 — Additional file 5 Table S5. Pre-processing of WGBS data using MethylStar increases the number of high-confident cytosines that can be used for epimutation analysis compared with previous pre-processing approaches. [file 13059_2020_2161_MOESM5_ESM.pdf]

Table S5

MA	coverage > 3 AllC	CG	CHG	CHH	posteriorMax >=0.99 AllC	CG	CHG	CHH
**MA1_1**	13417233	2344610	2513665	8558958	23428047	3030753	3797292	16600002
**MA1_3**	25009841	3450911	3937253	20731677	29141823	3753716	4183152	21204955
**MA3**	25679815	3086050	3542387	19051378	24357974	3655356	4131622	16570996

(yellow)	**Previous methods**
(blue)	**MethylStar**

Table S5: Pre-processing of WGBS data using MethylStar increases the number of high-confident cytosines that can be used for epimutation analysis compared with previous pre-processing approaches.
