# Supplementary material for: AlphaBeta: computational inference of epimutation rates and spectra from high-throughput DNA methylation data in plants
Source: Genome Biol. 2020 Oct 6;21:260. doi: 10.1186/s13059-020-02161-6 (PMC7539454; doi:10.1186/s13059-020-02161-6)
Supplement: Supplementary file 6 — Additional file 6 Figure S1. Developmental origin of somatic epimutations in plants. [file 13059_2020_2161_MOESM6_ESM.pdf]

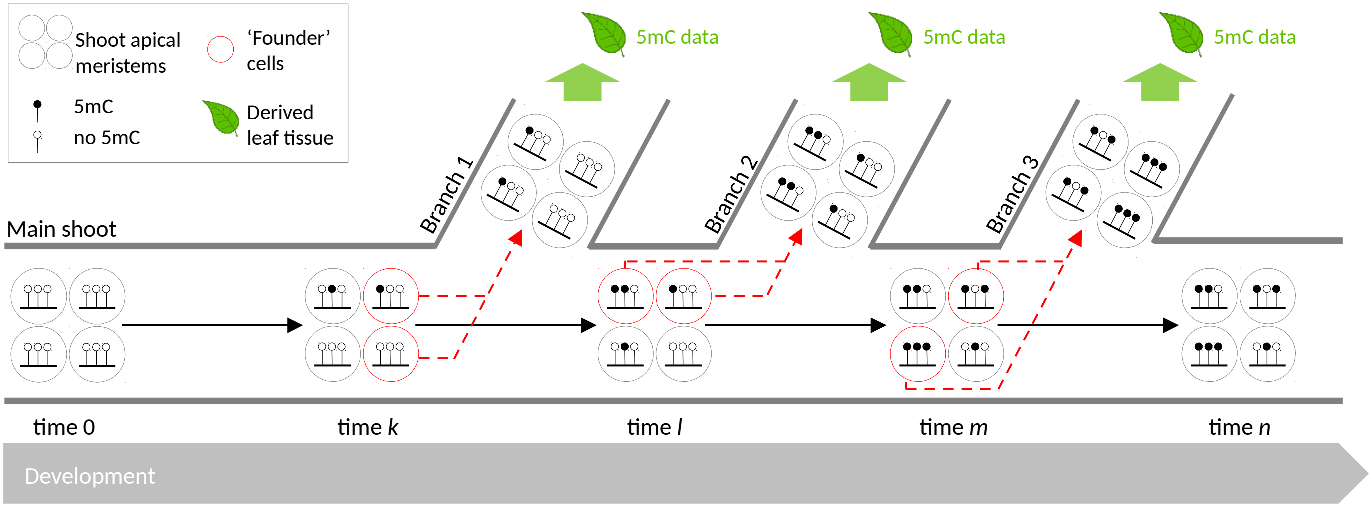

Fig. S1: **Developmental origin of somatic epimutations in plants.** The failure to maintain the methylation status of cytosines during the mitotic maintenance of shoot apical meristematic cell pools leads to spontaneous somatic epimutations. Shown here are only spontaneous gains of methylation, for simplicity. A small set of 'founder' cells gives rise to lateral branches at developmental times *k*, *l*, and *m*. The random sampling of founder cells creates a bottleneck which increases the frequency of somatic epimutations in the cell populations of lateral branches. Somatic epimutation accumulation in shoot apical meristems thus leads to increased 5mC divergence between leafs originating from different lateral branches (e.g. leaf methylomes from Branch 1 and 2 are more similar than those from Branch 1 and 3).
