# Supplementary material for: AlphaBeta: computational inference of epimutation rates and spectra from high-throughput DNA methylation data in plants
Source: Genome Biol. 2020 Oct 6;21:260. doi: 10.1186/s13059-020-02161-6 (PMC7539454; doi:10.1186/s13059-020-02161-6)
Supplement: Supplementary file 7 — Additional file 7 Review history. [file 13059_2020_2161_MOESM7_ESM.docx]

Review History

**First round of review
Reviewer 1**

**Were you able to assess all statistics in the manuscript, including the appropriateness of statistical tests used? Yes, and I have assessed the statistics in my report.**

**Were you able to directly test the methods?** No

**Comments to author:**

I have carefully gone through these two manuscripts "The somatic genetic and epigenetic mutation rate in a wild long-lived perennial Populus trichocarpa" and "AlphaBeta: Computational inference of epimutation rates and spectra from high-throughput DNA methylation data in plants" (referred to as Paper 1 [Hofmeister et al.] and Paper 2 [Shahryary et al.] hereafter). Due to the close relationship between theses twos, I would like to give my comments as a whole for the two papers.

Both papers focus on epigenetic variation in the ancestors and their progenies or in the age-different branches from the same tree. The epimutation variation (roughly as DNA methylation used in these papers) has been widely studied among different stages of development in a plant, various organs of a plant, and the individual plants under different environments. Similarly, these two studies proposed the pedigree-based or age-based variation of epimutation. Generally speaking, the epimutation variation are influenced by many factors so little studies have revealed clear patterns with biological significance. If such clear patterns could be obtained between pedigree (or age) and epimutation, those studies could be very important scientifically.

These two studies have made positive progress on such patterns. For example, they provide evidences to show neutral pattern of epimutation accumulation in context CG in generation by generation in a pedigree and age-increased branches. Because this pattern is present in all analyzed species and in both pedigree- and age-related samples, they conclude this pattern is deeply conserved in plant, which can be used as a molecular clock for age-dating trees. The significant accumulation of epimutation (basically the 5mC methylation) as time increase provides insight to understand epimutation variation. However, the biological significance is not so clear and such patterns revealed by these studies are not so strong.

Comments on both papers:

1) Consequences of the neutral pattern of epimutation accumulation. The most striking finding claimed by authors is the neutral pattern which is conserved in plant species. (e.g., in Discussion: "One of most striking findings was the close similarity in the epimutation landscapes between these very different systems" in page 21). Based on this finding, epimutation is expected to accumulate much more when a branch was separated much earlier from the others. In fact, all new leave in the branches of the tree 13 should be equal separated to all the samples from tree 14. Therefore, the comparison between the two trees is an extreme contrast which should result in much more accumulation of epimutations. Similarly, when the epimutation accumulates as generation increases, a logical inference is that the epimutation accumulation should be much more between two close-related (which relationship can be easily identified by a phylogenetic tree) individual plants in a population (e.g., different Arabidopsis individuals from the same accession), because the two plants

obviously came from a common ancestor plant with hundreds or thousands of generations. This comparison should be very easy to do and should produce much more strong patterns. These kinds of comparisons between extreme-samples should provide much more robust results if the pattern revealed by these papers are real.

2) These two manuscripts overlap to some extent. The Paper 1 develop a new bioinformatics tool and use poplar data to test for its robustness. However, many of the results about mutation and epimutation coincide with previous reports and may not be unexpected. So, I suggest the authors are better to consider combining these two manuscripts into one.
3) Biological significance of epimutation accumulation. Based the results in these studies, epimutation accumulation will lead to DNA methylation diversity. If it is true, the DNA methylation diversity (or epimutation) is somewhat equivalent to DNA diversity (or mutation) which will also accumulate as generation by generation or by increased age. Two kinds of diversity (mutations) could have some common features. Is it possible to discuss a few sentences to give some possible biological significance?

Comments specifically on Paper2 [Shahryary et al.]:

This is a co-submitted paper with another interesting paper that I have reviewed. The presented work is also of fundamental important and is very useful. I need to be honest that I'm not an expert in mathematics modeling so I would leave most of the mathematic works to other experts. At least I see no big flaws in most of the inferences.

Some more minor issues:

Page 5 Line 116 (I use the line number in right side). What's the lower boundary of "sufficiently large" in real experiments?

1) Page 9 Line 207. "... prevent the formation of stable epimutations, ..."; Line 29 "..., independently of RdDM"
2) Organization of the Results. There are 13 sub-titles of results in Paper 1. Six of them starts as Analysis of and the other six begins as Robust estimates of, Estimate or Inferring. Too many sub-titles and too many similar sub-titles reflect the lack of logical organization for Results. As a good paper, the Results should focus on the core aims of a study rather than show the results one by one from one species to another. The results should be re-organized based on importance of results to the core aims.
3) Page 15 Line 380-Page21 Line 543. This whole part is a duplicate of Line 216-Line 379
4) The citation is poorly formatted as the journal title is missing. Also, it seems that many key references came from the same group (authors themselves). I suggest to cite more papers from different studies which will give more confidence for readers like me.

**Reviewer 2**

**Were you able to assess all statistics in the manuscript, including the appropriateness of**

**statistical tests used?** Yes, and I have assessed the statistics in my report.

**Were you able to directly test the methods?** No
**Comments to author:**

The authors developed AlphaBeta as a bioinformatic tool to stimate epimutation rates and spectra from pedigree-based methylomes, given the topology of the pedigree is known. The tool can be also used in the somatic epimutation rates in long-lived perennials. The tool was tested on Arabidopsis and dandelion and a poplar tree that was submitted back to back. The method itself is very helpful to the community, and the results from testing the method are interesting. My concerns are that how different are this AlphaBeta from the authors' published methods on PNAS 2015. In addition, since this is a method paper, it would be more helpful to describe the method (e.g., equations and models) more clearly to the readers in general), rather to focus on the data analysis. Also the story of poplar should go to the co-submitting manuscript or combine the 2 co-submitting manuscripts.

The context of figures (e.g., 1A, 1B, 2A...) should be explained in main text to improve readability, which is the reason of having figures. It is also helpful to simplify the figures. Long and over-complicated legends should go to Methods or Supplemental.

Is AlphaBeta simply an implementation of a previously published method of authors (van der Graaf et al 2015 PNAS)? What are the novelty and significant differences between AlphaBeta and the PNAS paper, to justify the publication on GB?

As this is a method/software paper, the main text should be on explaining the method it self. Testing on real datasets is needed but not the main point (other wise this should be another bio-data analysis manuscript).

Are there similar tools or methods that are used for estimating age epimutation rates? The authors should compare AlphaBeta with those models/tools on the same datasets.

What are the possible reasons for non-CG methylation with neutral epimutations only restricted in the large pedigrees? Why is the testing data not powered enough?

Page10-13. the whole polar story should be in either the co-submitting paper or here but not both. These paragraphs here look like the supplementary information that should go with the poplar manuscript.

The reference format should follow the journal format to include the publication year and journal names,

**Reviewer 3**

**Were you able to assess all statistics in the manuscript, including the appropriateness of**

**statistical tests used?** Yes, and I have assessed the statistics in my report.

**Were you able to directly test the methods?** Yes
**Comments to author:**

The authors report a new tool named AlphaBeta that looks to use a model based approach to investigate epimutation rates using high thoughput DNA methylation. The tool allows a user to investigate epimutation rates in respect to 4 different models, ABneutral, ABmm, ABuu, ABnull. ABneutral assumes spontaneous 5mC gains, ABmm assumes selection against loss of 5mC, ABuu assumes selection against gains and ABnull is the null model, e.g. no accumulation. The main finding from the application of the tool is that close similarities were observed in the per-generation CG epimutation rates between A. thaliana, dandelion and popla and that all operate under the ABneutral model.

Not being an expert in the area of methylation results within plants it is not possible for me to comment on the novelty of the finding. The tool is available from bioconductor and has ok documentation but it would be useful to include better documentation on the structure of the .fn file which appears to be a custom file format rather than ask users to work it out from an existing example file. I would also suggest some documentation on how some of the figures in this manuscript could be produced using the tool.

I also don't agree with the authors that they have identified neutral divergence in a CHH or CHG context as reported in Figure 3C. The results are confined to a single experiment (for the most part e.g. excluding the promoter comparison) and this experiment while having the largest number of samples seems to have the most variability (Figure 3D). Conversely you could argue that MA1_3 and MA3 replicate at the global level an ABnull model.

The CHG ABneutral model in poplar looks more convincing it would be useful to see a similar table as to S2-S4 for this analysis. It also seems strange the authors don't focus on this result. The authors also seem to make little comment on this, including in the discussion.

I would also appreciate a comment on Figure 3D. There seems to be an initial increased rate of Divergence which then seems to become linear. Can the author explains why their model produces this initial higher rate and whether a linear model would provide a better fit?

Minor Comments:

The divergence as defined in equation 1 seems to be equivalent to the mean absolute difference of methylation between 2 samples but the authors seem to use a complicated indicator function which overcomplicates it.

P11 line 19-20: prevent the formation sta- ble epimutations "Missing an of"

P11 line 29-30: that maintains methylation states at a subset of CHG and CHH sites, independently RdDM -> Needs an of?

**Authors Response
Reviewer #1:**

This is a co-submitted paper with another interesting paper that I have reviewed. The presented work is also of fundamental important and is very useful. I need to be honest that I'm not an expert in mathematics modeling so I would leave most of the mathematic works to other experts. At least I see no big flaws in most of the inferences.

Some more minor issues:

1) Page 5 Line 116 (I use the line number in right side). What's the lower boundary of "sufficiently large" in real experiments?

**We have removed this ambiguous sentence. The determination of sample size would require a thorough power analysis of MA-pedigree designs in conjunction with our inference method. This would involve – among other things – an *empirical* assessment of how the measurement of 5mC divergence (D) is affected by different NGS approaches (e.g. WGBS, RRBS, epiGBS), sequencing depths, growth conditions, propagation method (e.g. clonal versus sexual), and sampling schemes (e.g. progenitor vs. sibling of progenitor). In addition, it would be important to know how total divergence time (delta t) between samples from the pedigree impacts epimutation rate inference. Future studies could/should explore this problem systematically. This would yield answers to questions such as “What is the *minimum* number of samples needed to detect significant epimutation accumulation with a rate *as low as* W, given NGS approach X, sampling scheme Y and MA-design Z. That such a power analysis is currently lacking does not undermine our findings, but it does – as correctly pointed out by this reviewer - put into question our statement on page 5, line 116.**

2) Page 9 Line 207. "... prevent the formation of stable epimutations, ..."; Line 29 "...,

independently of RdDM"

**We have corrected this.**

3) Organization of the Results. There are 13 sub-titles of results in Paper 1. Six of them starts as Analysis of and the other six begins as Robust estimates of, Estimate or Inferring. Too many subtitles and too many similar sub-titles reflect the lack of logical organization for Results. As a good paper, the Results should focus on the core aims of a study rather than show the results one by one from one species to another. The results should be re-organized based on importance of results to the core aims.

**We apologize for the confusion. Our original submission had several textual formatting issues related to Latex to PDF conversion. One issue was the duplication of several paragraphs which this reviewer also spotted (see point 4 below). The other issue was that the headers of ‘sections’ and ‘sub-sections’ appeared at the same level, which made it difficult to discern the structure/logic of our results. We had issued a corrected version of our original submission to the editors, but apparently this version did not make it into the hands of this reviewer in time. We apologize for this.**

**As can be seen in our revised manuscript, the “Results” section (now called “Application”) is organized by major experimental system: selfing-derived MA-lines (A. thaliana), asexually-**

**derived MA-lines (dandelion) and development/aging (poplar). The mathematics (i.e. inference approach) underlying these experimental systems differs substantially. Our goal was to show that/how *AlphaBeta* can be applied to such diverse systems, and guide readers who may have generated such mutation accumulation data to the correct application of our method. In addition, we introduced sub­sections within each experimental system that highlight selected insights into spontaneous epimutations in plants. In response to this reviewer’s comments, we made an effort to re-word the headers of these sub-sections in order to capture – more accurately - the novelty of each finding. Our choice for highlighting certain biological findings was to tell a story that would connect the different experimental systems.**

4) Page 15 Line 380-Page21 Line 543. This whole part is a duplicate of Line 216-Line 379 **We have corrected this.**
5) The citation is poorly formatted as the journal title is missing. Also, it seems that many key references came from the same group (authors themselves). I suggest to cite more papers from different studies which will give more confidence for readers like me.

**We have added several references related to this topic.**

**Reviewer #2:**

1. The authors developed AlphaBeta as a bioinformatic tool to estimate epimutation rates and spectra from pedigree-based methylomes, given the topology of the pedigree is known. The tool can be also used in the somatic epimutation rates in long-lived perennials. The tool was tested on Arabidopsis and dandelion and a poplar tree that was submitted back to back. The method itself is very helpful to the community, and the results from testing the method are interesting. My concerns are that how different are this AlphaBeta from the authors' published methods on PNAS 2015. **Our PNAS 2015 publication presented epimutation rate estimates in selfing-derived MA-lines of *A. thaliana*. In that paper, we developed a neutral epimutation model, and fitted this model to multi-generational WGBS data. Model inference was implemented via in-house C++ code. The code was never released to the end-users, as the focus of this paper was on the biology. The present paper makes substantial new contributions on the methodological and software side. Below we are highlighting these contributions:**

1. **New models:**

**In addition to a neutral epimutation model, we have now developed selection models that allow for statistical tests of neutrality. These new models enable us to assess if spontaneous DNA methylation gains and losses are under positive or negative selection, and thus provide novel insights into the forces that act on epimutational processes over time. Moreover, we have also extended our mathematical models to mutation accumulation systems that are obtained via clonal/asexual propagation and to the developmental analysis of trees.**

2. **New estimation procedure:**

**Our previous inference approach relied on a brute-force grid search over a four-dimensional parameter space. For reasonable grid sizes and resolution, these models would run several days to obtain a single estimate. For the *AlphaBeta* software we have implemented a nonlinear optimization routine using the R optimx library, which provides estimates within seconds. The implementations of this estimation procedure was not trivial.**

3. **User-friendly software:**

**The *AlphaBeta* software is released through Bioconductor, and thus connects to the popular**

**Bioconductor and R user-ecosystem. Since the software is open source it can also be flexibly**

**improved by the end-user. Finally, the package is supported by a solid and evolving manual, which should increase the reach of our software.**

4. **Standard data inputs and plotting utilities:**

**An important aspect of the new software is its ability to read and interpret any arbitrary pedigree topology from relatively simple input files. To make this possible, we treat the pedigree as a sparse, directed network and use existing network analysis packages in R for importing, exporting, plotting and manipulating the network. Using these utilities we can convert any pedigree or phylogeny into the data structure that is necessary for epimutation rate inference.**

**In our view, the above points constitute a substantial advance over our previous method, not only methodologically but also in terms of the biological questions that can be addressed.**

2. In addition, since this is a method paper, it would be more helpful to describe the method (e.g., equations and models) more clearly to the readers in general), rather to focus on the data analysis. Also the story of poplar should go to the co-submitting manuscript or combine the 2 co-submitting manuscripts.

**We have re-structured the paper. The centerpiece of the manuscript is now a detailed description of the method in the main text (see section ‘The AlphaBeta method’), followed by its various applications. This new format places more emphasis on the development of the actual method, rather than on the data analysis.**

3. The context of figures (e.g., 1A, 1B, 2A...) should be explained in main text to improve readability, which is the reason of having figures. It is also helpful to simplify the figures. Long and over-complicated legends should go to Methods or Supplemental.

**We agree with this reviewer that Figures 1A and 1B may have been superfluous. To ease readability we have completely removed Figure 1B, and have moved Figure 1A to SI. The new Figure 1 (former Figure 2) provides a conceptual overview of the *AlphaBeta* software package. Since the description of the *AlphaBeta* method has now been given more priority in the text, we think that reference to this conceptual figures is sufficiently motivated and also useful to support several statements/explanations in the text.**

Is AlphaBeta simply an implementation of a previously published method of authors (van der Graaf et al 2015 PNAS)? What are the novelty and significant differences between AlphaBeta and the PNAS paper, to justify the publication on GB?

**See response response to Point 1.**

As this is a method/software paper, the main text should be on explaining the method it self. Testing on real data sets is needed but not the main point (other wise this should be another bio-data analysis manuscript). **We have re-structured the paper to focus more on the method itself (see response to Point 2.).**

Are there similar tools or methods that are used for estimating age epimutation rates? The authors should compare AlphaBeta with those models/tools on the same data sets.

**There are currently no alternative tools for estimating epimutation rates from pedigree-based WGBS data. Several population genetic models have been adapted to account for the unique properties of epimutations (e.g. high (epi)mutation rates relative to the effective genome size, violation of infinite sites assumption, frequent reversibility, rate asymmetry) (see Wang & Fan 2015, Jiang et al, Charlesworth and Jain 2014, Vidalis et al. 2016). The latter methods can be applied to WGBS data from natural populations and have been used to analyze the so-called methylation site frequency spectrum (mSFS) of *A. thaliana* natural ecotypes. Although useful,**

**these methods cannot be applied to pedigree type of data, and also yield no direct estimates of the actual per site, per haploid genome epimutation rates. Hence, *AlphaBeta* is truly unique in its capabilities.**

What are the possible reasons for non-CG methylation with neutral epimutations only restricted in the large pedigrees? Why is the testing data not powered enough?

**We know from plotting 5mC divergence (D) against divergence time (delta T) that epimutation accumulation in non-CG contexts is either absent or very slow. The biological reasons for this are still unclear, but one possibility is that these loci are selectively targeted for methylation re-enforcement by the RNA directed DNA methylation (RdDM) pathway. On the statistical side, the lack of accumulation (or the detection thereof) may stem from a combination of low epimutation rates for non-CG sites and the the increased WGBS measurement error that is known to exist for these contexts. Rejecting the null hypothesis of no accumulation requires us to test whether the epimutation rate (i.e. the accumulation slope) is significant different from zero. The power of this test is essentially a function of sample size.**

**As we have pointed out to Reviewer 1, the determination of sample size would require a thorough power analysis of MA-pedigree designs, in general, in conjunction with our inference method. This would involve – among other things – an *empirical* assessment of how the measurement of 5mC divergence (D) is affected by different NGS approaches (e.g. WGBS, RRBS, epiGBS), sequencing depths, growth conditions, propagation method (e.g. clonal versus sexual), and sampling schemes (e.g. progenitor vs. sibling of progenitor). In addition, it would be important to know how total divergence time (delta T) between samples from the pedigree impacts epimutation rate inference. Future studies could/should explore this problem systematically. This would yield answers to questions such as “What is the *minimum* number of samples needed to detect significant epimutation accumulation with a rate *as low as* W, given NGS approach X, sampling scheme Y and MA-design Z. Such analyses would certainly be useful but are beyond the scope of the present manuscript. Accordingly, we have down-tuned our statements regarding our detection of CHH epimutation accumulation in pedigree MA1-1 and added the sentence:**

“However, the ability to consistently detect these accumulation patterns from multi-generational pedigree data should be explored more systematically in future studies, particularly as a function of sample size (M), divergence time (delta T) and measurement uncertainly in 5mC divergence (D).”

Page10-13. the whole polar story should be in either the co-submitting paper or here but not both. These paragraphs here look like the supplementary information that should go with the poplar manuscript. **Upon consultation with the editor, we have been asked to resubmit both manuscripts as originally submitted.**

The reference format should follow the journal format to include the publication year and journal

names

**We have corrected this.**

**Reviewer #3:**

The authors report a new tool named AlphaBeta that looks to use a model based approach to investigate epimutation rates using high throughput DNA methylation. The tool allows a user to investigate epimutation rates in respect to 4 different models, ABneutral, ABmm, ABuu, ABnull. ABneutral assumes spontaneous 5mC gains, ABmm assumes selection against loss of 5mC, ABuu assumes selection against gains and ABnull is the null model, e.g. no accumulation. The main finding from the application of the tool is that close similarities were observed in the per-generation

CG epimutation rates between A. thaliana, dandelion and poplar and that all operate under the ABneutral model.

Not being an expert in the area of methylation results within plants it is not possible for me to comment on the novelty of the finding. The tool is available from bioconductor and has ok documentation but it would be useful to include better documentation on the structure of the .fn file which appears to be a custom file format rather than ask users to work it out from an existing example file.

**We thank this reviewer for his/her comment. Our input files with extension .fn are actually simple text files with no special structure. The reason why we introduced the .fn extension was to be able to automatically detect these files in a folder that may also contain other (possibly temporary) files with extensions such as .csv or .txt. The use of the .fn extension also allowed us to read the input files, irrespective of the actual file names the user may have given them.**

**However, this reviewer’s comment prompted us to reflected more deeply on the type of files and file structure we would like to use as input to *AlphaBeta*. A challenge in developing data import functionalities for our software has been the question how we can reconstruct the topology of any arbitrary pedigree from relatively simple input files (i.e. files that the user can easily generate manually and without error) . We have now solved this problem by treating the pedigree as a sparse, directed network. This enabled us to use existing network analysis packages in R for importing, plotting and manipulating the pedigrees. Our input files now use the standard "nodelist" and "edgelist" file format that is standard for the R igraph package. The input formats are now discuss in detail here** [**https://github.com/jlab-code/AlphaBeta/blob/**](https://github.com/jlab-code/AlphaBeta/blob/) **master/vignettes/AlphaBeta.pdf. Using these new utilities we can now convert any pedigree or phylogeny into the data structure that is necessary for epimutation rate inference. Realizing that the preparation of input files and their import is an important and essential step in the entire analysis workflow, we have also substantially improved the corresponding documentation in the user manual (see above link).**

I would also suggest some documentation on how some of the figures in this manuscript could be produced using the tool.

**We have included now several plotting functions in the updated Bioconductor package, which generate several useful plots similar to those shown in the manuscript. These plotting functions make use of utilities from the *igraph* package in R for plotting pedigrees/phylogenies with arbitrary topologies. In addition, we have included functions for potting delta T versus D along with the model fits. These later functions lean on the popular *gg plots* library, and thus allow the R user to customize plots for their own needs. These functions act directly on the *AlphaBeta* output files. The user manual now provides several plotting examples.**

I also don't agree with the authors that they have identified neutral divergence in a CHH or CHG context as reported in Figure 3C. The results are confined to a single experiment (for the most part e.g. excluding the promoter comparison) and this experiment while having the largest number of samples seems to have the most variability (Figure 3D). Conversely you could argue that MA1_3 and MA3 replicate at the global level an ABnull model.

**We agree that the evidence for neutral epimutation accumulation in context CHH and CHG is not very strong, given that statistical support for this effect only comes from pedigree MA1_1 (all annotations) and also in pedigree M3 (TEs and promoters). However, our hypothesis tests do reject the null of no accumulation and it is coincidental that these two pedigrees are the largest in terms of sample sizes. We feel that it was particularly important to highlight the insight on CHH, because epimutation rate estimates in this context had never been performed and it was categorically assumed that the stable formation of epimutations at CHH sites**

**cannot occur, as these sites are targeted by the *de novo* RNA-directed DNA methylation (RdDM) pathway. In addition to our statistical conclusions, we also show for the first time that many of the CHH sites that show epimuation accumulation in the MA-lines are actually not targeted by RdDM but by the maintenance methylstransferase CHROMOMETHYLASE 2 (CMT2), an enzyme that maintains methylation at a subset of CHG and CHH sites. This latter observation provides a possible molecular explanation for the accumulation of CHH epimutations over generation time, at least for specific CHH sub-contexts. In the revised version we have expanded this biological insight. At the same time we have tried to down-tune our statistical conclusions. This new paragraph reads:**

“The detected accumulation of CHH epimutations was somewhat surprising, given that cytosine methylation in this context is typically targeted by the RNA-directed DNA methylation pathway (RdDM). The de novo action of this pathway should prevent the formation of trans-generationally stable epimutations, particularly those originating from DNA methylation loss (Teixeira et al. 2009). To explore this observation in more detail we inspected specific CHH sites that showed stable methylation status changes over generation time (Fig. 2E). Our analysis revealed that these CHH sites actually fall outside of known sRNA clusters and are therefore unlikely involved in RdDM (Fig. 2F). Instead, they appear to be targeted by CHROMOMETHYLASE 2 (CMT2), an enzyme that maintains methylation at a subset of CHG and CHH sites, independently RdDM. Support for this hypothesis comes from the fact that these CHH sites are enriched for trinucleotide context CWA (W = A, T) (Fig. 2G), which is a preferred substrate for CMT2 binding (Gouil et al. 2016). Moreover, a re-analysis of a *cmt2* methylation mutant from Stroud et al. (2013) revealed a marked reduction in cytosine methylation at these CHH sites relative to wt (Fig. 2H), providing additional evidence for a maintenance role of CMT2 at these loci.

Taken together, these results provide a possible molecular explanation for the accumulation of CHH epimutations over generation time, at least for specific CHH subcontexts. However, the ability to consistently detect these accumulation patterns from multi-generational pedigree data should be explored more systematically in future studies, particularly as a function of sample size (M), divergence time (delta T) and measurement uncertainly in 5mC divergence (D).”

The CHG ABneutral model in poplar looks more convincing it would be useful to see a similar table as to S2-S4 for this analysis. It also seems strange the authors don't focus on this result. The authors also seem to make little comment on this, including in the discussion.

**We have added a comment regarding the accumulation of CHG epimutation in the main text. We agree that a full result table equivalent to S2-S4 is useful. We are providing such a table in our co-submitted paper.**

I would also appreciate a comment on Figure 3D. There seems to be an initial increased rate of Divergence which then seems to become linear. Can the author explains why their model produces this initial higher rate and whether a linear model would provide a better fit?

**5mC divergence over time is a function of two processes: 1. the segregation and fixation of stable epiheterozygote loci that originate from the pedigree founder; 2. the generation of new epimutations in independent lineages through *de novo* epimutations via the rates alpha and beta. The first process accounts for the rapid and highly non-linear increase during the first ~ 8 generations (this process has been well described in the theoretical analysis of classical lines crosses, see below). The second processes accounts for the continued, nearly linear increase thereafter. Our model captures both of these processes and we have already commented on this effect in van der Graaf et al (2015). However, to reiterate this important point, we have rewritten part of this section:**

“We started by plotting genome-wide (global) 5mC divergence (D) against divergence time (deltaT). D increases as a function of deltaT in all pedigrees (Fig. 2D). A characteristic pattern is the rapid, non-linear increase in D for the first ~ 8 generations followed by a nearly linear increase. As pointed out before [12], the initial non-linearity is driven by the stable segregation and fixation of epiheterozygote loci that originate from the pedigree founder, a phenomenon that has been well-described in the classical theory of experimental line crosses (Fisher 1949, Broman 2012, Johannes 2011, Bartlett 1935). By contrast, the subsequent linear increase in D is mainly due to the accumulation of new epimutations that arise de novo during inbreeding. The co-occurence of these two processes is restricted to mutation accumulation systems that are propagated sexually. In clonally- or asexually-derived MA-lines, the non-linear increase in D should be absent, as can indeed be seen in our later analysis of poplar and dandelion (see below).“

Minor Comments:

The divergence as defined in equation 1 seems to be equivalent to the mean absolute difference of methylation between 2 samples but the authors seem to use a complicated indicator function which overcomplicates it.

**Indeed, this equation simply describes the mean absolute differences, and we now explicitly say this. However, we still think it is useful to present this equation in terms of the indicator function because it makes explicit how the divergence is calculated from the actual DNA methylation state calls (i.e. the input data), which is introduced in the preceding paragraph. In response to the other reviewers we have now re-structured our paper and moved the mathematical description of the method more centrally into the main text. In the context of this (now) more technical section, the use of the indicator function make more sense.**

P11 line 19-20: prevent the formation stable epimutations "Missing an of"
**Corrected.**

P11 line 29-30: that maintains methylation states at a subset of CHG and CHH sites, independently

RdDM -> Needs an of?

**Corrected.**

**Second round of review**

**Reviewer 1**

Reviewer 1 has no further concerns.

**Reviewer 2**

Thanks for the responses to my comments.
**Reviewer 3**

I thank the authors for there extensive efforts in responding to my review and appreciate the great improvement in the documentation for the package, including the effort to utilise existing data structures for input allowing other tools to easily integrate as necessary.
